# Managing Temporomandibular Joint Osteoarthritis by Dental Stem Cell Secretome

**DOI:** 10.1007/s12015-023-10628-9

**Published:** 2023-09-26

**Authors:** Maria Bousnaki, Athina Bakopoulou, Ioannis Grivas, Chrysa Bekiari, Andreas Pich, Marta Rizk, Kleoniki Keklikoglou, Eleni Papachristou, Georgios C. Papadopoulos, Aristeidis Kritis, Antonios G. Mikos, Petros Koidis

**Affiliations:** 1 Department of Prosthodontics, School of Dentistry, Faculty of Health Sciences (FHS), Aristotle University of Thessaloniki (AUTh), 54124 Thessaloniki, Greece; 2 Department of Anatomy, Histology & Embryology, School of Veterinary Medicine, Aristotle University of Thessaloniki (AUTh), 54124 Thessaloniki, Greece; 3 Research Core Unit Proteomics &, Institute of Toxicology, Hannover Medical School, 30625 Hannover, Germany; 4 Department for Preventive Dentistry, Periodontology and Cariology, University Medical Center Göttingen, 37073 Göttingen, Germany; 5 Institute of Marine Biology, Biotechnology and Aquaculture (IMBBC), Hellenic Centre for Marine Research (HCMR), Thalassocosmos, P.O. Box 2214, 71003 Heraklion, Crete Greece; 6 Biology Department, University of Crete, 70013 Heraklion, Crete Greece; 7 Department of Physiology and Pharmacology, School of Medicine, Faculty of Health Sciences (FHS), Aristotle University of Thessaloniki (AUTh), 54124 Thessaloniki, Greece; 8 Department of Bioengineering, Rice University, Houston, TX 77030 USA

**Keywords:** Temporomandibular Joint, Osteoarthritis, Dental pulp stem cells, Secretome, In vivo

## Abstract

The potential therapeutic role of the Dental Pulp Stem Cells Secretome (SECR) in a rat model of experimentally induced Temporomandibular Joint (TMJ) Osteoarthritis (OA) was evaluated. Proteomic profiling of the human SECR under specific oxygen tension (5% O2) and stimulation with Tumor Necrosis Factor-alpha (TNF-α) was performed. SECR and respective cell lysates (CL) samples were collected and subjected to SDS-PAGE, followed by LC-MS/MS analysis. The identified proteins were analyzed with Bioinformatic tools. The anti-inflammatory properties of SECR were assessed via an in vitro murine macrophages model, and were further validated in vivo, in a rat model of chemically-induced TMJ-OA by weekly recording of the head withdrawal threshold, the food intake, and the weight change, and radiographically and histologically at 4- and 8-weeks post-treatment. SECR analysis revealed the presence of 50 proteins that were enriched and/or statistically significantly upregulated compared to CL, while many of those proteins were involved in pathways related to “extracellular matrix organization” and “immune system”. SECR application in vitro led to a significant downregulation on the expression of pro-inflammatory genes (MMP-13, MMP-9, MMP-3 and MCP-1), while maintaining an increased expression of IL-10 and IL-6. SECR application in vivo had a significant positive effect on all the clinical parameters, resulting in improved food intake, weight, and pain suppression. Radiographically, SECR application had a significant positive effect on trabecular bone thickness and bone density compared to the saline-treated group. Histological analysis indicated that SECR administration reduced inflammation, enhanced ECM and subchondral bone repair and regeneration, thus alleviating TMJ degeneration.

**Graphical Abstract:**

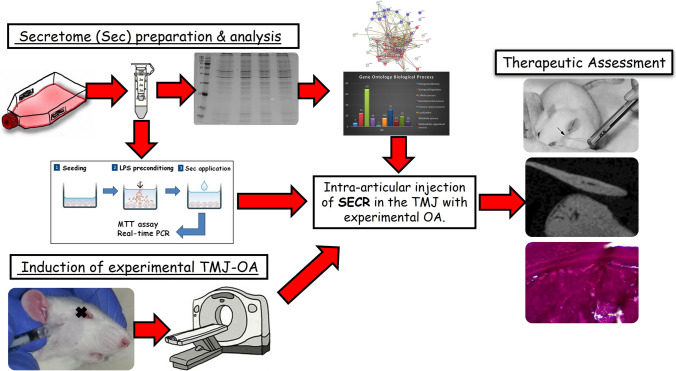

**Supplementary Information:**

The online version contains supplementary material available at 10.1007/s12015-023-10628-9.

## Introduction

Temporomandibular Joint (TMJ) Osteoarthritis (OA) is a chronic, low-inflammatory, degenerative disease [1] manifested with pain, inflammation of synovial membrane, gradual cartilage degradation, and subchondral bone remodeling [2]. The etiology is multifactorial and still unclear with several factors implicated with its establishment and progression, identifying low-grade inflammation as the principal [1]. Plethora of modalities exist advocating management of TMJ-OA, targeting most on relieving pain, increasing mandibular range of motion, and decreasing inflammation [3]. However, to foster and dream of managing the disease and alleviate the destructive process, there is necessity to unravel, in the name of new-old concepts, the intrinsic regulatory network linking homeostasis, macromolecules, metabolism, and inflammation [4]. TMJ-OA in this respect could be considered a “dissipative structure/system”[5], with fluctuating entropy in the course of inflammation, periodically out of equilibrium, from a near homogenous to an inhomogeneous state, strangling for a breakdown with therapeutic effect, thus regenerative medicine along with stem cells (SCs) and by-products emerge as a tentative revolutionary strategy to tailor deviation of reaction rates and diffusion [6].

Mechanisms through which mesenchymal stem cells (MSCs) promote tissue regeneration and their therapeutic potential have not been currently implicated to in situ differentiation following cell transplantation but, to the trophic factors secreted by the MSCs, namely the MSC secretome (MSC-SECR). The latter represents the sum of cytoprotective soluble factors produced and secreted by the MSCs [7]. These include growth factors, chemokines, cytokines and hormones, with paracrine effects in cell differentiation, matrix synthesis, angiogenesis, tissue repair and immunomodulation [7], thus, instead of directly transplanting MSCs, MSC-SECR or MSC-derived extracellular vesicles (EVs) are used [8]. Based on a recent systematic review paper on the applications of the MSC-SECR in preclinical models of experimentally-induced OA, resolution of inflammation and cartilage regeneration were the main effects of its application implying its validity in OA management [9]. However, most of the studies evaluated MSC-SECR application in models of knee OA, therefore, restricting direct extrapolation for TMJ-OA, with two studies evaluating MSC-SECR application in TMJ-OA models, with promising outcomes [10, 11].

Cell-free approaches such as those involving the MSC-SECR have become more popular than cell-based treatments due to ease of processing, lower manufacturing cost, storage, non-immunogenicity, and non-tumorogenicity [12]. Cell-free therapies are more time- and cost-effective avoiding expansion and maintenance of cultured MSCs, offering off-the-shelf therapies to manage acute conditions, and eliminating safety issues associated with cell transplantation, such as immune compatibility.

Dental pulp stem cells (DPSCs) present an alternative choice to MSCs due to their great clinical potential, easy accessibility, and minimally invasive harvesting [13]. DPSCs have exhibited promising potential in cartilage regeneration [14], while also showing high affinity towards regenerating tissues of the craniofacial region since they share the same embryological origin [15].

Proteomic profiling of human DSPCs secretome—(SECR) under variable microenvironmental conditions, such as oxygen tension (normoxia-20% vs. hypoxia 2%) and/or stimulation with Tumor Necrosis Factor alpha (TNF-α), followed by in vitro validation revealed that SECR was substantially enriched with anti-inflammatory, tissue repair and regenerative factors [16].

Based on the above, it was the aim of this study to evaluate whether SECR application would result in the alleviation of inflammation in an in vitro inflammation model through the reduction of proinflammatory cytokines, as well as the improvement in clinical parameters, the improvement on the trabecular bone thickness and bone density radiographically, and enhanced ECM and subchondral bone repair histologically in the in vivo experimental rat model of TMJ-OA.

## Materials and Methods

### Study Design

The objective was to optimize SECR from DPSCs under specific culture conditions, which was then followed by assessment in vitro and in vivo in inflammation and experimental OA models, respectively. hDPSCs were isolated from extracted third molars and exposed to hypoxia (5% O_2_) with parallel stimulation with 10 ng/ml tumor necrosis factor-alpha (TNF-α) for 24 h. SECR was collected and processed for SDS-page electrophoresis followed by LC–MS/MS analysis. The therapeutic effects were assessed utilizing MTT cell viability assay and real-time RT-PCR analysis at an in vitro model of inflammation with RAW macrophage-like cells. SECR was then applied intra-articularly in a rat model of chemically induced TMJ-OA. 1wk (T0) before induction of TMJ-OA (Τ1, baseline) animals were evaluated for clinical parameters, such as HWT, food intake and weight (Suppl. Fig. 5). More specifically, 0.5 mg of MIA dissolved in 50 μl of PBS was injected in the right TMJ. The experimental TMJ-OA was induced in all animals on the right TMJ, while left TMJ served as control (no intervention) (naive control TMJ). There were two groups (N = 21 per group); the first group received arthrocentesis and saline wash (OA + saline; sham control), while the second arthrocentesis, saline wash, and SECR injection (OA + SECR; experimental group) (group A: 50 µl saline, group B: 50 µl saline + 50 µl concentrated SECR). Animals were randomly allocated in each group using block randomization. Treatment was initiated 4wks after MIA-injection, when TMJ-OA was established, according to the clinical assessment (Head Withdrawal threshold measurement). The treatment protocol was applied weekly, for a total of 3 times. Additionally, four animals received intra-articular injection of MIA in both TMJs (*N* = 8 TMJs) to induce experimental OA, and those animals represented the OA group (positive control), and were euthanized after 4 weeks; while 4 animals were monitored weekly without any intervention, and those animals represented the naïve control group in evaluating the clinical outcomes of the study i.e. food intake and weight.

Clinical measurements were performed every week, and prior to treatment application. Four and 8wks after the initiation of treatment, the animals were euthanized (*N* = 21 at 4wks, 10 from group A and 11 from group B, and *N* = 21 at 8wks, 11 from group A and 10 from group B) by means of transcardiac perfusion with 0.9% saline, followed by 4% paraformaldehyde (PFA) solution in PBS at pH = 7.4. Then, both TMJs of each animal were surgically removed, fixed in 4% PFA solution in PBS at pH = 7.4 overnight and processed for radiographic evaluation by means of μCT; then the same samples were further used for histology and immunohistochemistry. More specifically, samples were stained with Hematoxylin & Eosin (H & E) and Alcian Blue (AB), and joint repair quality was evaluated using the OARSI grading system and the Mankin's histological scoring (Tables 1 and 2). Additionally, samples were stained for COLI (NB600-408), VEGF (AF-493-NA) and von Willebrand factor (ab6994) and were observed under a fluorescence/confocal microscope. Necessary actions were taken to minimize bias, as both the caregiver and investigator applied the treatments and assessed the outcomes, were blinded.

**Table 1:** Mankin's histological scoring

Criteria	Score	Histological finding
Structure	0	Smooth intact surface
	1	Slight surface irregularities
	2	Pannus/surface fibrillation
	3	Clefts into transitional zone
	4	Clefts into radial zone
	5	Clefts into calcified zone
	6	Total disorganization
Cells	0	Uniform cell distribution
	1	Diffuse cell proliferation
	2	Cell clustering
	3	Cell loss
Toluidin staining	0	Uniform staining
	1	Minor discoloration
	2	Moderate discoloration
	3	Severe discoloration
	4	Total discoloration
Tidemark integrity	0	Intact
	1	Vascularity

**Table 2:** OARSI grading system

OA cartilage histopathology grade assessment		
Grade (key feature)	Subgrade (optional)	Associated criteria (tissue reaction)
Grade 0: surface intact, cartilage intact	No subgrade	Intact, uninvolved cartilage
Grade 1: surface intact	1.0 Cells intact	Matrix: superficial zone intact, edema and/or fibrilation
	1.5 Cell death	Cells: proliferation (clusters), hypertrophy, reaction must be more than superficial fibrillation only
Grade 2: surface discontinuity	2.0 Fibrilation through superficial zone	As above
	2.5 Surface abrasion with matrix loss within superficial zone	+ Discontinuity at superficial zone ± Cationic stain matric depletion (Safranin O or Toluidine Blue) upper 1/3 of cartilage (mid zone) ± Disorientation of chondron columns
Grade 3: vertical fissures	3.0 Simple fissures	As above
	3.5 Branched/ complex fissures	± Cationic stain matric depletion (Safranin O or Toluidine Blue) into lower 2/3 of cartilage (deep zone) ± New collagen formation (polarized light microscopy, Pico Sirius Red stain)
Grade 4: erosion	4.0 Superficial zone delamination	Cartilage matrix loss, cyst formation within cartilage matrix
	4.5 Mid zone excavation	As above
Grade 5: denudation	5.0 Bone surface intact	Surface is sclerotic bone or reparative tissue including fibrocartilage
	5.5 Reparative tissue surface present	As above
Grade 6: deformation	6.0 Join margin osteophytes	Bone remodeling. Deformation or articular surface contour (more than osteophyte formation only)
	6.5 Joint margin and central osteophytes	Includes: microfracture and repair
I. Grade = depth progression into cartilage		

### Culture of DPSCs and Collection and Processing of the Culture Conditioned Medium (SECR) and the Respective Cell Lysates (CL)

DPSCs were isolated from extracted third molars by the enzymatic dissociation method [17] and characterized for “stemness” by flow cytometry. More specifically, DPSCs have been characterized in terms of mesenchymal stem cell markers and chondrogenic differentiation potential in a previous study [18]. They were further assessed for adipogenic and osteogenic differentatiation potential (Supplementary material, Suppl. Fig. 1). Τhe study was approved by the Institutional Ethical Review Board (Protocol No. 20/29–11-2017) and patients signed an informed consent form.

DPSCs from the established cultures were seeded at 3 × 10^5^ cells/well in 6-well plates and cultured for 48 h with a-MEM (Minimum Essential Media) culture medium (Invitrogen), supplemented with 15% FBS (EU-tested, Invitrogen), 100 mM L-ascorbic acid phosphate (Sigma-Aldrich, Taufkirchen, Germany), 100 units/ml penicillin, 100 mg/ml streptomycin and 0.25 mg/ml Amphotericin B (all from Invitrogen) (= Complete Culture Medium- CCM) at 37 °C in 5%CO_2_. When DPSCs reached 70–80% confluence, were washed three times with phosphate buffered saline (PBS) and medium was changed to a-MEM without fetal bovine serum (FBS). SECR was collected from DPSC cultures exposed to hypoxia (5%O_2_) and parallel stimulation with 10 ng/ml tumor necrosis factor-alpha (TNFa) (PeproTech, London, UK) for 24 h. After the 24 h-stimulation, medium was changed to CCM, supplemented with protease inhibitor cocktail and the DPSCs were further cultured in the predefined hypoxic conditions for another 48 h. SECR samples were collected from three different biological replicates of the experiment and concentrated by approximately tenfold using the Corning SPIN-x concentrator (Sigma-Aldrich) with a 5000 MWCO cut-off, and centrifugated at 15,000 g for 15 min at 4 °C. The concentrated samples were collected, aliquoted, and stored at − 80 °C, until subjected to SDS-PAGE electrophoresis.

In parallel to the SECR collection, cell lysates (CL) from the respective DPSC cultures exposed to hypoxia (5% O_2_) and parallel stimulation with 10 ng/ml TNFa were also collected and further processed [16].

### LDH Assay

To assess any cytotoxic impact of the preconditioning methods on the DPSCs that could have resulted in plasma membrane damage, lactate dehydrogenase (LDH) presence into the SECR was measured utilizing the LDH assay (Pierce LDH Cytotoxicity Assay Kit, Thermo Fisher, Scientific).

### SDS-PAGE-Based Sample Fractionation and Trypsin Digestion

SECR and CL samples were mixed with 4 × Laemmli buffer (100 mM Tris/HCl, pH 8.8, 10%SDS, 100 mM DTT, 3%glycerol, 2 mg/ml bromophenol blue) and heated at 95 °C for 5 min. After the samples were cooled to RT, 2 μl of 40% acrylamide (Sigma-Aldrich) were added to alkylate cysteine residues, followed by incubation for 30 min at RT. Samples were then subjected to sodium dodecyl sulfate/polyacrylamide gel electrophoresis (SDS-PAGE). CL and SECR samples were loaded at 10% and 15% acrylamide separating gels, respectively, with a 5% stacking gel. After separation, proteins were visualized with Coomassie Briliant Blue R-250 (Bio-rad, Feldkirchen, Germany). Sample lanes were then cut into pieces of ≈1 mm^3^ and subjected to in‐gel digestion with trypsin (the whole lane has been processed in subfractions)[19]. Gel pieces were destained with 50% ACN/50 mM ammonium bicarbonate, dehydrated with 100% ACN, and dried by vacuum centrifugation [20]. Dried gel pieces were incubated in sufficient trypsin solution (9 μg/ml trypsin from Serva, 10% acetonitrile ACN, 50 mM ammonium bicarbonate) for 1 h on ice. After the addition of 150 μl of 10%ACN containing 20 mM ammonium bicarbonate, gel slices were incubated overnight at 37 °C. Digestion was stopped by adding 100 μl 50% ACN containing 2% trifluoroacetic acid (TFA). After incubation for 15 min at 37 °C, supernatants were collected. Peptides were further eluted from the gel slices in four consecutive steps (150 μl 50% ACN with 0.5% TFA, 60 min, 37 °C; then, 150 μl 50% ACN with 0.5% TFA, 60 min, 25 °C; and finally, 100 μl 100%ACN, 15 min, 20 °C, twice). Supernatants from all incubations were combined and dried in a vacuum centrifuge. Peptide extracts were re-dissolved in 30 μl 2% ACN, 0.1% TFA with shaking at 800 rpm for 20 min. After centrifugation at 20,000xg supernatant was directly analyzed with LC–MS or stored at -20 °C.

### LC-MS/MS Analysis

Peptides were analyzed by LC‐MS/MS, as previously described [16, 20]. Raw data were processed using Max Quant software (version 1.6.) [21], and reviewed human entries of the Uniprot database (UP000005640, May 2020). Protein identification was based on the default parameters used with Max Quant. Briefly, Mass accuracy was set to 4.5 ppm for precursor ions of charge 2 to 7 and 0.5 Da for MS-2 fragments obtained by ion trap MS. Oxidation at methionine, deamidation at aspartate and glutamate, acetylation at the N-terminal amino acid methionine, and alkylation by acrylamide at cysteine residues were used; at maximum five modifications were allowed for one peptide. Digestion with trypsin was allowed after all lysine and arginine residues, and two missed cleavages were accepted. Matching between runs was allowed using a match time window of 0.7 min. Search for second peptides was allowed. Identifications were based on a target/decoy strategy and a false discovery rate < 0.01 on peptide and protein level. Quantification was done with the Max Quant LFQ normalization algorithm, and intensity values were given as logarithm to the basis of 2. Data calculation was done using Perseus [22] and excel. To allow calculations, missing values were imputed using fixed values of 2^12, because most proteins should have not been quantified, due to very low concentration. With Perseus software testing options were used and all tests were controlled by p-values to identify significantly altered proteins.

### Bioinformatics Analysis

Gene Ontology (GO) (http://geneontology.org/) analysis and functional categorization of the identified proteins was performed through evolutionary relationships (PANTHER) classification system (http://www.pantherdb.org/tools/). By GO the proteins were mapped into the categories cellular compartment, biological process, and signaling pathways using a standardized vocabulary. Reactome Pathway Database (Version 72) was also used to identify protein involvement in biological pathways, while String (Version 11.0) software was used to visualize protein interaction networks (https://reactome.org/, https://string-db.org/). STRING constitutes a free database of known and predicted protein interactions, which also includes direct (physical) and indirect (functional) associations that are obtained from different sources (genomic context, high-throughput experiments, co-expression, and previous knowledge) [23].

### In Vitro Validation of the Anti-Inflammatory Effects of the SECR

RAW 246.7 cells (ATCC, VA, USA), were cultured with high glucose DMEM supplemented with 10% FBS and antibiotics (100U/ml penicillin and 100 mg/ml streptomycin) at 37 °C under humidified atmosphere of 5%CO_2_ and stimulated with 1 μg/ml LPS (L2143, Sigma Aldrich) for 4 h, followed by the application of the proposed concentrated SECR for 24 h. The therapeutic effects of SECR addition to this inflammation model were assessed utilizing the MTT cell viability assay and RT-PCR analysis.

### Cell Viability Assay

Cell viability/proliferation of the RAW 246.7 cells was determined using the MTT [3-(4, 5-dimethylthiazol-2-yl)-2,5-diphenyltetrazolium bromide] assay [16].

### Real Time RT-PCR

Total RNA was isolated and real-time RT-PCR was performed to quantitatively evaluate gene expression profiles [16]. Primers were designed using the PRIMER BLAST (https://www.ncbi.nlm.nih.gov/tools/primer-blast/) for the following genes: MMP8, MMP9, MMP13, IL6, IL10, MCP-1 (Table 3). A standard melting curve was used to check quality of amplification and specificity. Results were adjusted by amplification efficiency (LinRegPCR) and were normalized to 2 stable housekeeping genes, ACTβ and GAPDH, as evaluated by geNorm.

**Table 3:** Primers designed for the Real-time PCR analysis of several inflammatory and anti-inflammatory-related genes and the respective amplicon sizes of the PCR products

Primers designed for the Real-time PCR analysis of several inflammatory and anti-inflammatory genes and the respective amplicon sizes of the PCR products			
Gene symbol	Forward (5'-3')	Reverse (5'-3')	Amplicon size (bp)
MMP8	CAATGCCTTCCCAGTACCTGA	TTTTCGTAGGTAATTCTCAGCAGTT	70
MMP9	TGGTCTTCCCCAAAGACCTG	AGCGGTACAAGTATGCCTCTG	72
MMP13	ACCTTCTGGTCTTCTGGCAC	AAGTTGTAGCCTTTGGAACTGC	108
IL6	CTCTGCAAGAGACTTCCATCCA	AGTCTCCTCTCCGGACTTGT	92
IL10	CCAGGTGAAGACTTTCTTTCAAAC	TCCTGCATTAAGGAGTCGGT	72
MCP-1	GCTGTAGTTTTTGTCACCAAGCTC	ACCTTAGGGCAGATGCAGTT	145
ACTβ	CGCAGCCACTGTCGAGTC	GTCATCCATGGCGAACTGGT	96
GAPDH	GGGTCCCAGCTTAGGTTCAT	CCCAATACGGCCAAATCCGT	100

### Animal Experiments

The animal experiments were performed in accordance with the Guidelines for Animal Experimentation of the Veterinary Directorate (Nr. 406539(2361)/26-09-2018) and were based on the ARRIVE guidelines in full compliance with the 3Rs’ rule for animals in research [24].

Adult healthy Wistar Rats (male and female, 3-6mnths old, 180-280 g the females and 320-380 g the males) were used. The animals were housed in plastic cages (1 rat per cage), that were filled with wood chips bedding and maintained under pathogen-free conditions in a temperature-controlled room (23 ± 2 °C) under a fixed 12 h light/dark cycle in an accredited animal facility. The animals had ad libitum access to food and water. Based on sample size analysis (Supplementary Material), it was estimated that 42 laboratory animals (21 per group) should be used. Finally, 42 animals were used in total (4 in the pilot studies, 3 for the OA group, 3 died during the experimental procedures, and 32 in the experimental groups, with 16 animals per group, the smallest possible that can yield a SS result). Regarding the number of male and female rats in each group, 9 female and 7 male rats were in each group.

### Clinical Evaluation—Food Intake and Weight Measurement

The animals were monitored weekly regarding food intake and weight measurement along with their health status monitoring, to detect signs of distress, illness, or behavioral changes. The animals were weighted weekly, at a fixed time, and the percentage (%) of body weight change from the baseline (i.e., day of induction of TMJ-OA) was calculated for each animal based on the following equation [25]:

$$\%\;\mathrm C\mathrm h\mathrm a\mathrm n\mathrm g\mathrm e\;\mathrm i\mathrm n\;\mathrm b\mathrm o\mathrm d\mathrm y\;\mathrm w\mathrm e\mathrm i\mathrm g\mathrm h\mathrm t=\left(\mathrm{weight}\;\mathrm{on}\;\mathrm{week}\;\mathrm x-\mathrm{weight}\;\mathrm{on}\;\mathrm{baseline}\right)\times100/\mathrm{weight}\;\mathrm{on}\;\mathrm{baseline}$$

The food intake was also recorded weekly at a fixed time and the percentage (%) of food intake change was determined using a similar equation as the one used for body weight change.

Additionally, 4 healthy animals, without any intervention, were monitored weekly for 12wks to record changes in body weight and food intake, and those results are reported as the naive control group regarding the above-mentioned outcomes.

### Pain Behavioral Measurements—Head-Withdrawal Threshold (HWT)

Pain behavioral assessment was performed by evaluation of the mechanical hyper-nociception by means of HWT measurement. HWT was measured using the von Frey filaments, where the lowest force was initially applied, followed by gradual increase in the applied force, until a withdrawal response would be recorded [26]. The HWT recorded would be the lowest force applied that would elicit a withdrawal reflex for at least 3 times [26]. HWT measurements were performed weekly in both TMJs of all animals until the end of the experiment. HWT was calculated as a mean value per joint of at least 8 joints per group. Clinical evaluation was performed by a blinded investigator.

### Radiographic Evaluation

The dissected TMJ samples were then exposed to radiographic analysis by a high-resolution μCT scanner (SkyScan 1172, Bruker, Kontich, Belgium, and SkyScan 1272, Bruker, Kontick, Belgium, at the Hellenic Centre for Marine Research-HCMR and at the University Medical Center Göttingen, respectively). The ROI was selected to include the condylar head and was defined by anatomical landmarks (a straight line from the most anterior point to the most posterior part of the condylar head) (Suppl. Fig. 4), to be reproducible among samples of different dimensions. The samples were scanned at a voltage of 80 kV and a current of 124μA with an aluminium filter of 0.5 mm. Images were acquired at a pixel size from 3 to 13.78 μm for a half rotation of 180° and the exposure time was 1435 ms. Images were reconstructed into cross-section images using SkyScan’s NR exon software (Bruker, Kontich, Belgium) which employs a modified Feldkamp’s back-projection algorithm. 3D analysis was performed using the SkyScan’s CTAn software (Bruker, Kontich, Belgium) for bone mineral density (BMD), bone volume (BV), bone surface (BS), trabecular bone thickness (Tb.Th), trabecular bone separation (Tb.S), structure linear density, and trabecular bone surface density (BS/TV).

The osseous components of the TMJ—primarily the condylar head- were further evaluated radiographically as previously described [27]for radiographic features and morphological alterations (such as subcortical sclerosis, subcortical cyst, surface erosion, and generalized sclerosis), which are typical hallmarks for the diagnosis of TMJ-OA [NO_PRINTED_FORM] (Suppl. Fig. 4).

### Histological Evaluation

Upon completion of the radiographic evaluation, the fixed TMJ samples were processed for histological analysis. The samples were decalcified in Decal III (EDTA/Hydrochloric acid) (Atom Scientific LTD, UK) for 4–6 wks, followed by serial dehydration and embedment in paraffin for microtomy (MICROM, LabX, Canada). Serial sections were cut at 8–10 μm and stained with H & E for the evaluation of tissue morphology, and with AB for the evaluation of glycosaminoglycan deposition. An inverted microscope (Nikon Eclipse 80i microscope, Nikon Instruments Inc, Japan) was used to evaluate the stained sections. OA staging was performed employing the OARSI grading system and the Mankin's histological scoring system by two blinded independent observers [28].

### Immunohistochemical Analysis

Immunohistochemical (IHC) analysis was performed to evaluate the presence of COLI, VEGF and von Willebrand factor inside the constructs. In brief, sections were deparaffinized, rehydrated and then heat induced antigen retrieval was performed with the application of 0.01 M citrate buffer for 12 min. Subsequently, non-specific binding sites were blocked with blocking buffer containing 5% Bovine Serum Albumin (BSA) and 1% Triton in PBS for 30 min. After washing, the primary antibodies were applied, and samples were incubated overnight at 4 oC. The primary antibody for COLI (rabbit polyclonal, Novus Biologicals) was used at a 1:100 dilution, for VEGF (goat polyclonal, RandD) was used at a dilution 1:13, and the primary antibody for von Willebrand factor (rabbit polyclonal, abcam), was used at a dilution 1:100. After 3 washes with PBS, the samples were incubated with the secondary antibodies in a working buffer with 2% BSA in PBS for 1 h in the dark. The secondary antibodies (mouse IgG or 1:200 rabbit IgG, both from Biotium, Hayward, CA, USA) were diluted at 1:200. The samples were washed twice with PBS, mounted with mounting medium containing phenylamide and glycerol and observed by means of a microscope (Leica Microsystems, Wetzlar, Germany), equipped with special fluorescence filters and a confocal microscope (Nikon EZ-C1 CLSM) and analyzed with the software Ez-C1-3.20.

### Statistical Analysis

Statistical analysis of data employed one- or two-way analysis of variance (ANOVA), while multiple comparisons between groups was performed with Tukey’s post-hoc test (LDH assay, qPCR, HWT, weight and food intake, μCT measurements, and histological assessment) and Sidak’s multiple comparisons post-hoc test (MTT assay) using the Prism 8.0 Software (GraphPad, CA, USA) (statistical significance for *p* < 0.05). Data were expressed as means ± standard deviation (SD). All data for Mankin score, OARSI score, nociceptive response, food intake and weight assessment, and μCT analyses were expressed as mean ± SD. SS was set as *p* < 0.05.

## Results

### In Vitro Experiments

#### Morphological Evaluation of DPSCs after Preconditioning

Morphological evaluation of DPSCs showed that preconditioning with 10 ng/ml TNF-α for 24 h in a microenvironment of serum deprivation and 5% hypoxia extended for 48 h affected cell morphology. The developed monolayer was less dense, while cells acquired a more elongated morphology compared to the control (Suppl. Fig. 2A a, b).

#### Cytotoxicity Assessment of Preconditioning

Cytotoxicity assessment revealed that the preconditioning method applied in DPSC cultures combining application of TNF-α and hypoxia did not have a cytotoxic effect as shown by the LDH assay (Suppl. Fig. 2B).

#### Proteomic Characterization of the SECR

**Fig. 1:**
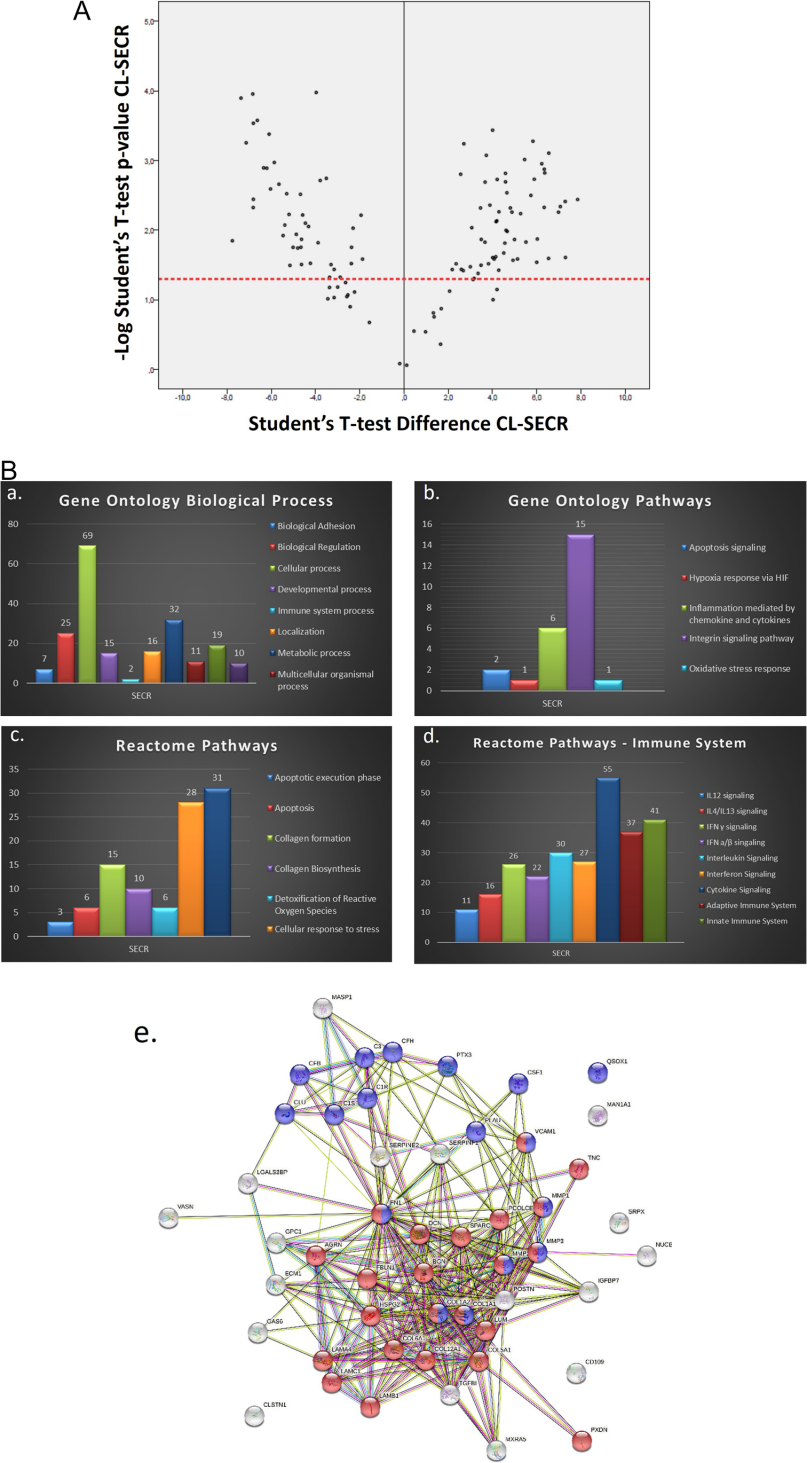
**A**. Quantitative proteomic analysis. Volcano plot reflecting the results from the statistical analysis of the comparisons between SECR and cell lysates (CL) samples. Dots located above the red dashed line represent proteins that are significantly differentially expressed. **B**. Gene ontology analysis. Gene Ontology (GO) analysis of the SECR samples regarding the term “Biological Process” (a), “Pathway” (b), and Reactome Pathway analysis of the SECR samples regarding several pathways (c) and pathways related to Immune System (d). Protein interaction network between the quantified proteins from SECR samples were generated. The String software was employed to perform the analysis on SS proteins from SECR involved in the pathways of interest from Reactome software from the comparisons of the CL against the SECR samples. These pathways included the “immune system” (blue nodes) and “extracellular matrix organization” (red nodes)

The proteome of DPSCs under specific preconditioning was analyzed. Protein extracts were separated with SDS-PAGE and analyzed by LC–MS/MS analysis. A protein had to be identified in 2 of the 3 replicate experiments to be included in the analysis. A total of 123 proteins were detected in the SECR. Comparative analysis from the SECR and the respective CL samples indicated that from the 123 proteins detected, 43 proteins were significantly enriched in the SECR, whereas 58 were significantly enriched in the CL (Fig. 1A).

#### Bioinformatic Analysis- GO- and Reactome-Based Analysis of the SECR

Cellular compartments, protein classes, and pathways of identified proteins were determined with GO analysis (Fig. 1B). Analysis regarding “biological process” revealed that most proteins were identified primarily under the term “cellular process”, followed by “biological regulation” and “metabolic process” (Fig. 1B a), while regarding “pathways” showed enrichment of the SECR in proteins related to “inflammation mediated by chemokine” and “integrin signaling pathway” (Fig. 1B b). To further investigate the different pathways of identified proteins’ involvement, samples were analyzed using the Reactome Pathway database, demonstrating that these proteins were involved in several pathways of interest, such as “cellular response to stress”, “detoxification of Reactive Oxygen Species (ROS)”, “extra-cellular matrix (ECM) organization”, “apoptosis”, and “collagen biosynthesis and formation”, as well as pathways related to the immune system (Fig. 1B c, d).

Protein interaction network between the quantified proteins from SECR samples were generated and are presented in Fig. 1B e. The String software was employed to perform the analysis on statistically significant (SS) proteins from the SECR samples involved in the pathways of interest from Reactome software from the comparisons of the CL against the SECR samples. These pathways included the “immune system” (blue nodes) and “extracellular matrix organization” (red nodes).

#### In Vitro Validation of the Anti-Inflammatory Effects of the SECR

The validated in vitro LPS-induced RAW 246.7 macrophage model was used to evaluate the anti-inflammatory action of the pre-conditioned SECR. Normally, the RAW 246.7 cells display a rather irregular, stellate morphology with multiple pseudopodia (Suppl. Fig. 3A a). LPS application (Suppl. Fig. 3A b) affected RAW 246.7 cells, which acquired a rounded morphology 24 and 48 h after LPS application (Suppl. Fig. 3A c, d). This effect was partly and totally reversed 24 h and 48 h respectively after by the application of the SECR (Suppl. Fig. 3A e, f).

The MTT assay showed that application of the SECR had a stimulatory effect on RAW 246.7 cell viability/proliferation at all tested time-points, resulting in SS increase in cell viability compared to control RAW 246.7 cells (*p* < 0.0001) (Suppl. Fig. 3B). This stimulatory effect was SS against LPS (*p* < 0.0001) after 72 h.

**Fig. 2:**
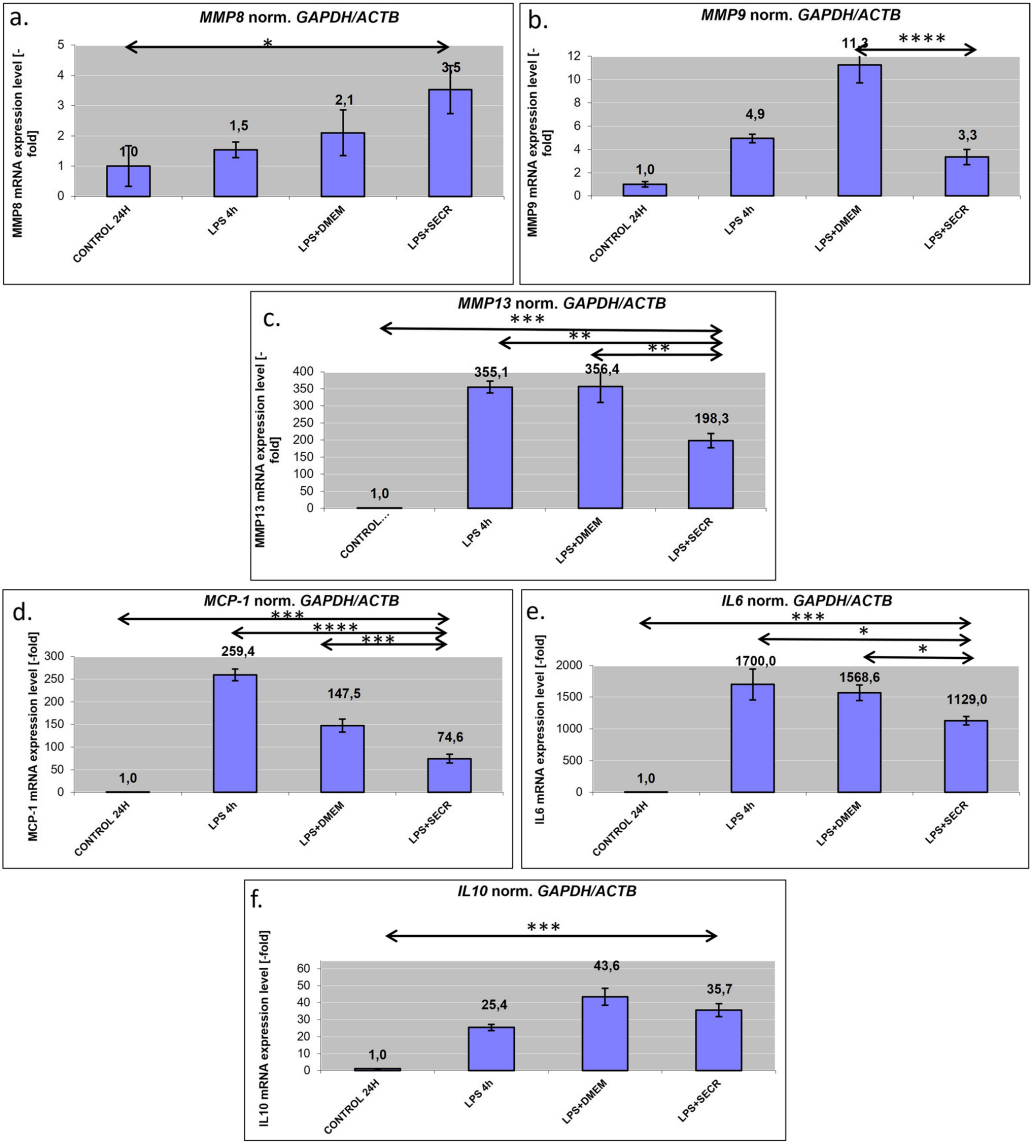
RT-PCR analysis. RT-PCR analysis of RAW cells after the application of lipopolysaccharide (LPS) and SECR for specific inflammatory (metalloproteinase (MMP)-8, **a**, MMP-9, **b**, MMP-13, **c**, and monocyte chemoattractant protein (MCP)-1), **d**, and anti-inflammatory markers (interleukin (IL)-6, **e**, and IL-10, **f**). *p < 0.05, **p < 0.01, ***p < 0.001, ****p < 0.0001

An analysis of the expression of several inflammation-related markers at the mRNA level revealed that the application of SECR on LPS-stimulated RAW 246.7 cells induced SS downregulation on the expression of pro-inflammatory genes, such as MMP-13, MMP-9, and MCP-1, while maintaining an increased expression of IL-10 and IL-6 (Fig. 2). Application SS downregulated the expression of MMP-13 (*p* = 0.004), and MCP-1 (*p* < 0.0001), compared to the LPS control samples (Fig. 2). Furthermore, RAW 246.7 cells exposed to SECR maintained an increased expression of IL-10 (Fig. 2).

### In Vivo Experiments

#### Clinical and Behavioral Evaluation

**Fig. 3:**
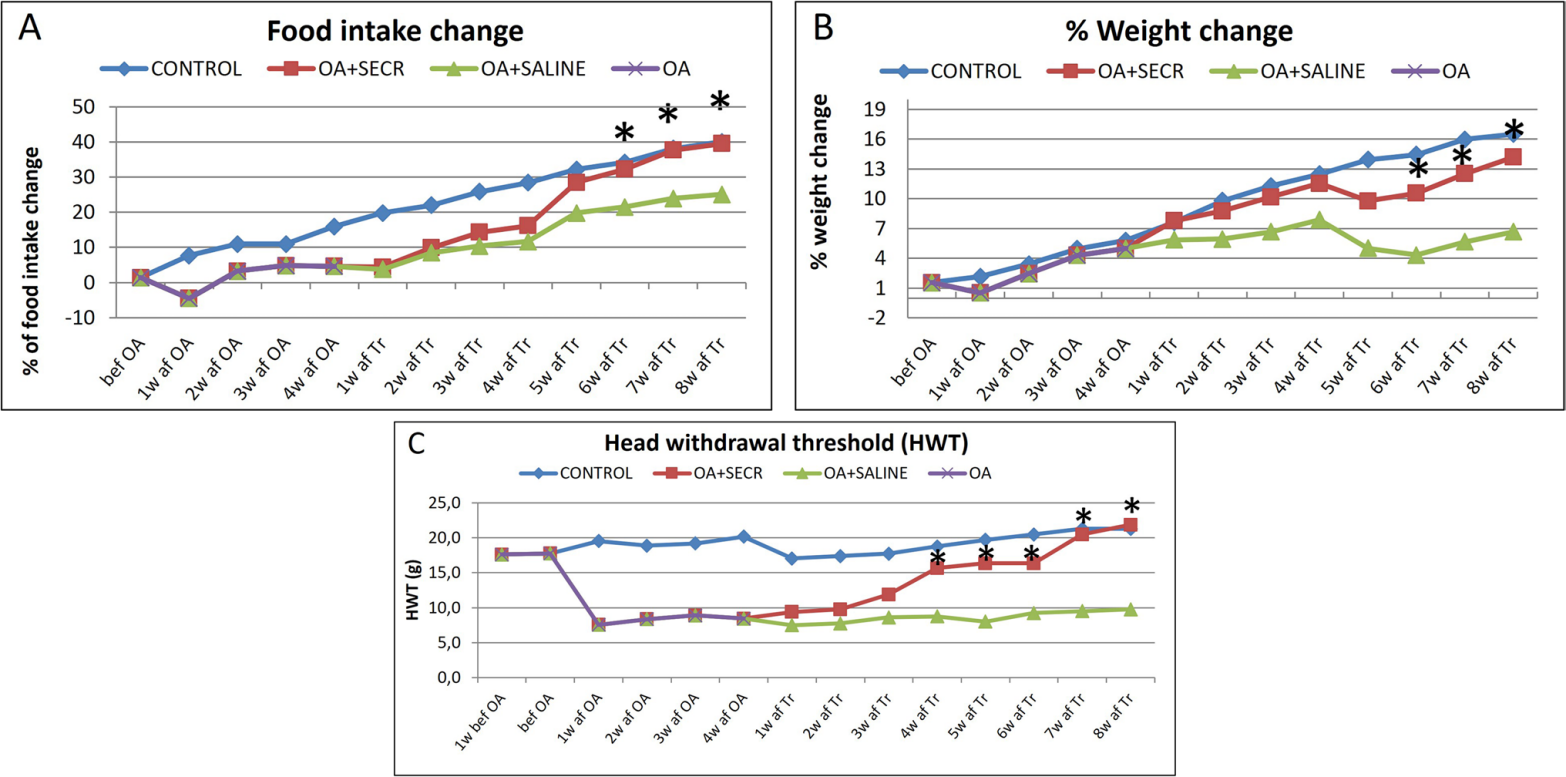
**A**. Food intake change percentages. Time-dependent food intake change percentages (%) following treatment of experimental TMJ-OA with SECR or saline. Treatment with the SECR had a SS positive effect on food intake outcome compared to the saline treatment at the 6th week post-treatment (*p* = 0.0434). **B**. Weight change percentages. Time-dependent weight change percentages (%) following treatment of experimental TMJ-OA with SECR or saline. A SS (*p* = 0.0277) increase in the % weight change was observed at the 6th week post-treatment in the OA + SECR group as compared to the OA + Saline group. **C**. HWT measurements. Time-dependent nociceptive responses following treatment with SECR. HWT was measured before MIA-induced TMJ-OA, after the induction of TMJ-OA, and during the time-course of treatment. HWT values significantly increased in the OA + SECR compared to the OA + Saline group from the 4wk post treatment (*p* < 0.0001), until the end of the experimental period (*p* < 0.0001)

Data analysis was not performed based on sex, because due to the randomization process, there was not a sufficient number of animals of each sex in each time point for the analysis. Clinical evaluation revealed that intra-articular injection of the optimized SECR in the affected right TMJ of the experimental (OA + SECR) group had positive effect on food intake outcome, which was evident from the 1wk post-treatment (Fig. 3A). Furthermore, treatment with the SECR had a positive effect on food intake outcome compared to the saline treatment (Fig. 3A). The difference between the two groups started increasing at 5wk post-treatment and reached SS at 6wk (*p* = *0.0434*) and was sustained until the end of experimental period. Additionally, from the 5^th^ week until the end the experimental (OA + SECR) group presented values of % food intake change that were close to those of the naïve control group (no SS) (Fig. 3A).

A SS increase (*p* = 0.0277) in the % weight change was observed at the 6^th^ week in the OA + SECR group as compared to the OA + Saline group (Fig. 3B). This difference further increased until the end of the experimental period, while there was no SS difference in the % weight change between the OA + SECR group and the naïve control throughout the experiment.

Along with the increase in food intake and positive weight change, SECR treatment had a positive effect on Head withdrawal threshold (HWT) values, which are the outcome of head withdrawal measurements after the application of pressure on the periauricular area over the TMJ. As depicted in the diagram in Fig. 3C, HWT gradually improved after the initiation of treatment in the SECR-treated joints, reaching levels similar to the naïve control joints after 7wks, whereas the saline-treated joints presented minimal improvement in HWT values throughout the course of the treatment. There was no SS difference in the HWT values between OA + SECR group and naïve control from the 4wk post-treatment until the end of experimental period. HWT values SS increased in the OA + SECR compared to the OA + Saline group from the 4wk post treatment (*p* < 0.0001), until the end of experimental period (*p* < 0.0001) (Fig. 3C).

#### Radiographic Evaluation – Results of the Micro-Computed Tomography Analysis

**Fig. 4:**
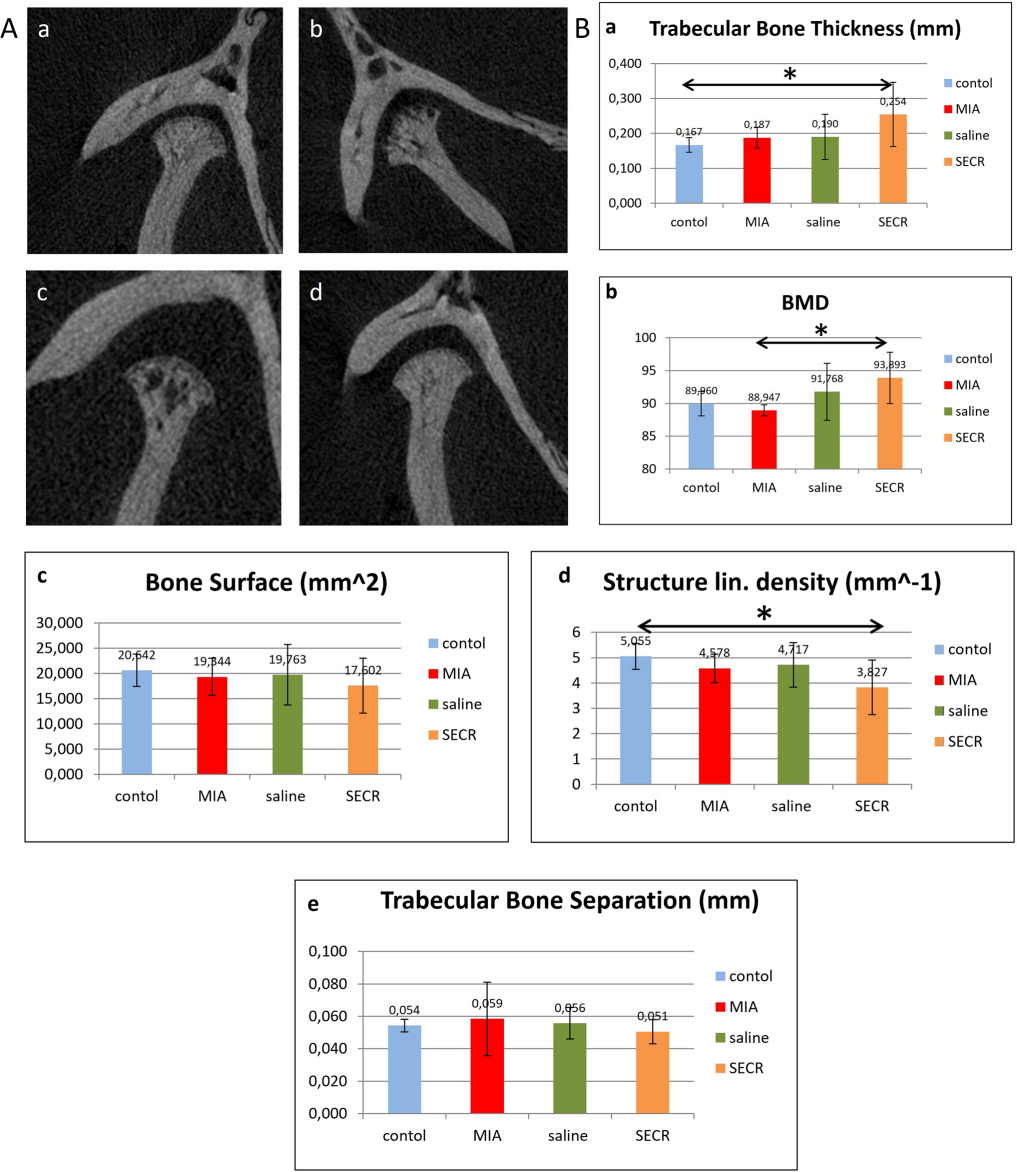
**A** μCT images after 4 weeks. Coronal view of μCT images of control TMJ (a), TMJ with MIA-induced OA (b), saline-treated TMJ after 4wks of treatment (c), and SECR-treated TMJ after 4wks of treatment (d). **B**. Bone structural parameters assessed with μCT after 4 weeks. (a) Trabecular bone thickness, (b) bone mineral density (BMD), (c) bone surface, (d) structure linear density, and (e) trabecular bone separation. *p < 0.05

Clinical evaluation results are well-correlated with radiographic data. Figure 4A depicts representative images of TMJ sections after 4wk of treatment. It can be observed from the coronal TMJ images that the naïve control samples (Fig. 4A a) have intact subchondral bone with a smooth, continuous surface, whereas the surface bone from the monosodium iodoacetate (MIA)-induced TMJ-OA positive control samples (Fig. 4A b) are irregular, with multiple subchondral bone erosions and defects. The SECR-treated TMJ (Fig. 4A c) resembles the control joint with smaller pores, whereas the saline-treated TMJ (Fig. 4A d) presents larger pores and irregular surface, providing a radiographical image close to the untreated TMJ-OA sample.

Besides, quantitative analysis of the micro-Computed Tomography (μCT) images was performed, where the condylar head was selected as the region of interest (ROI) (Suppl. Fig. 4). Changes in bone structural parameters were investigated, such as BMD, trabecular bone thickness (Tb.Th), trabecular bone separation (Tb.S), bone volume (BV), bone surface (BS), BV/TV, structure linear density (analogous to trabecular number), and structure model index (SMI). From this analysis at 4wks post-treatment, it can be observed that joints from the OA + SECR group (*n* = 8) presented Tb.S values close to the control (Fig. 4B e), illustrating the repairing processes that have taken place. In addition, SECR -treated joints present SS higher values of Tb.Th compared to control joints (*p* < 0.05) (Fig. 4B a), and SS lower values of structure linear density compared to the control (*p* < 0.05) (Fig. 4B d), both being indicatives of sclerotic changes. SECR-treated samples also show substantial increase in BMD over the OA group (*p* < 0.05) (Fig. 4B b).

**Fig. 5:**
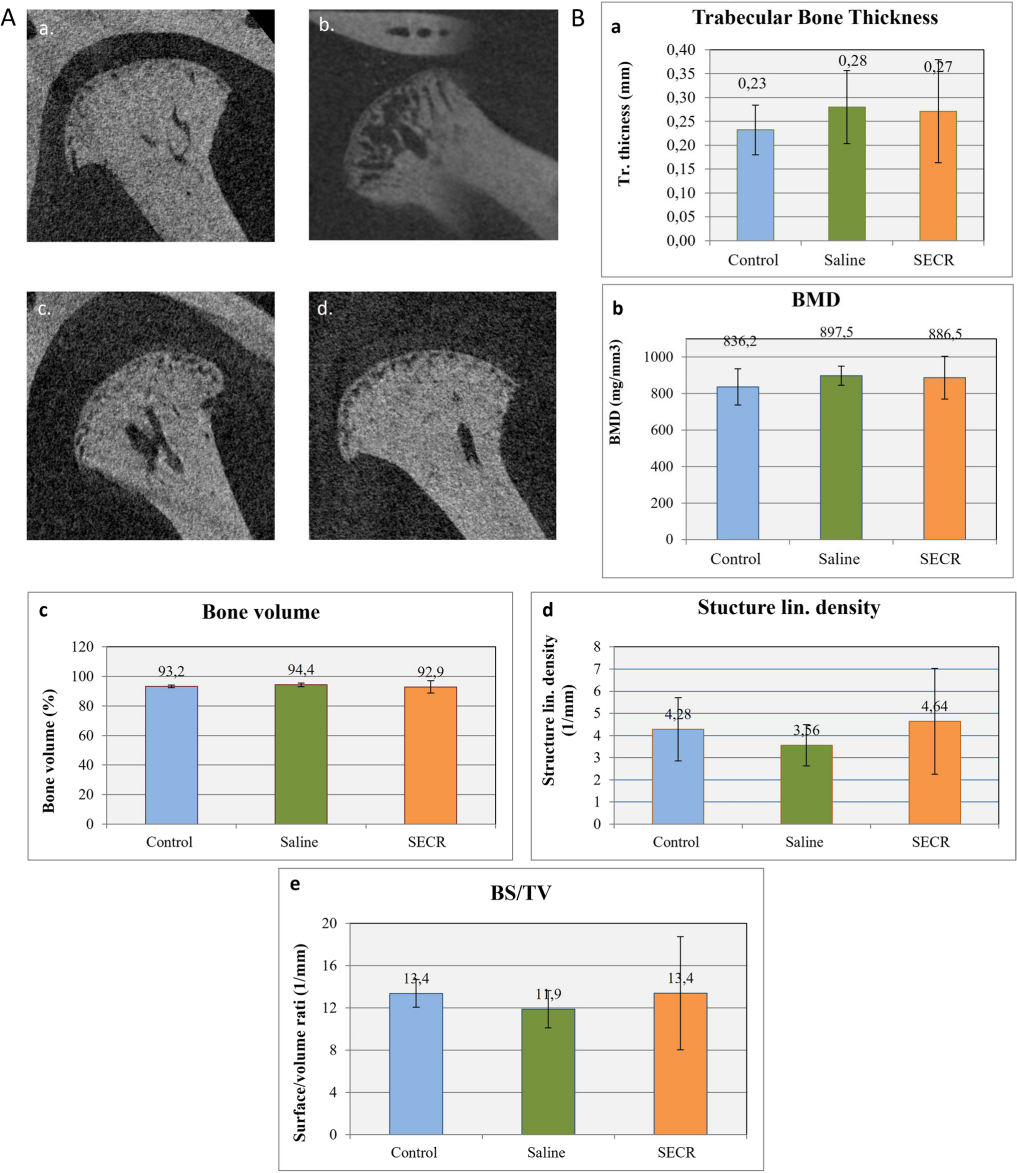
**A** μCT images after 8 weeks. Sagittal view of μCT images of control TMJ (a), TMJ with MIA-induced OA (b), saline-treated TMJ after 8wks of treatment (c), and SECR-treated TMJ after 8wks of treatment (d). **B**. Bone structural parameters assessed with μCT after 8 weeks. (a) Trabecular bone thickness, (b) bone mineral density (BMD), (c) bone volume, (d) structure linear density, and (e) bone surface density (BS/TV)

Quantitative analysis from the 8wk post-treatment samples showed that SECR-treated joints presented values similar to those of control samples regarding BV, structure linear density, and BS/BV, which is the volume of mineralized bone per unit volume of the sample, compared to the saline-treated samples; however, the differences among the two groups were not SS (Fig. 5).

The condylar head of TMJ was further analyzed for radiographic features and morphological alterations using criteria adopted by Ahmad et al. [29]. The most common alteration identified was subcortical cyst that refers to a cavity below the articular surface deviating from normal marrow pattern and was identified in 87.5% of the saline-treated samples 4wks post-treatment. Besides, surface erosion, referring to loss of articular surface continuity, was detected in 75% of the saline-treated samples. On the other hand, only 37.5% of the SECR-treated samples were identified with subcortical cyst, while there was no surface erosion identified in these samples at 4wks (Table 4).

**Table 4:** Radiographic assessment of condylar head morphological alterations following secretome or saline treatment

Timepoint	Groups	Subcortical sclerosis	Subcortical cyst	Surface erosion	Generalised sclerosis
4 weeks	Secretome (*n* = 8)	12.5%	37.5%	0	0
	Saline (*n* = 8)	0	87.5%	75%	0
8 weeks	Secretome (*n* = 4)	0	25%	0	0
	Saline (*n* = 4)	0	25%	25%	25%

#### Histological Evaluation

**Fig. 6:**
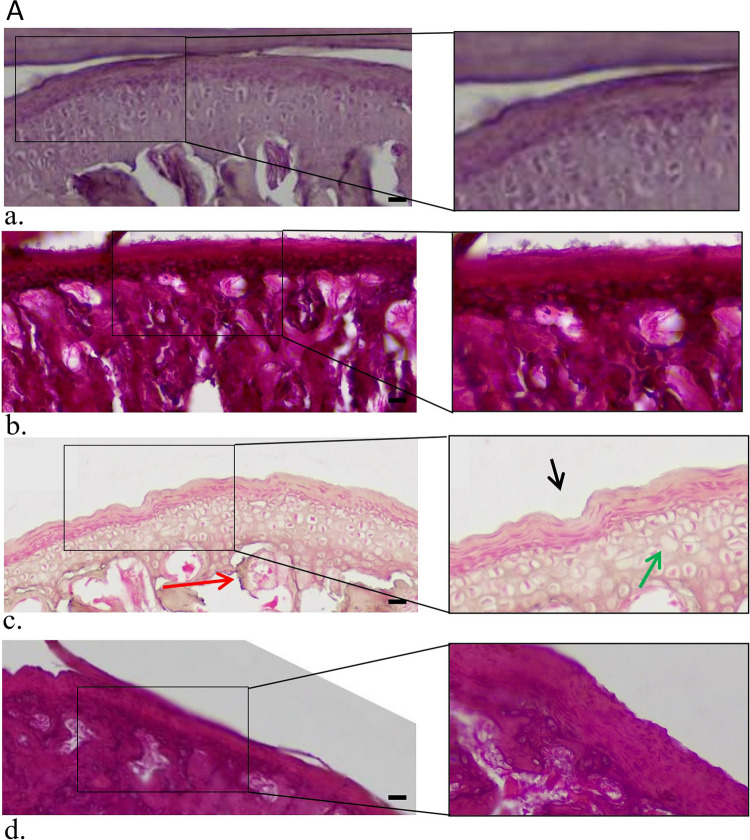

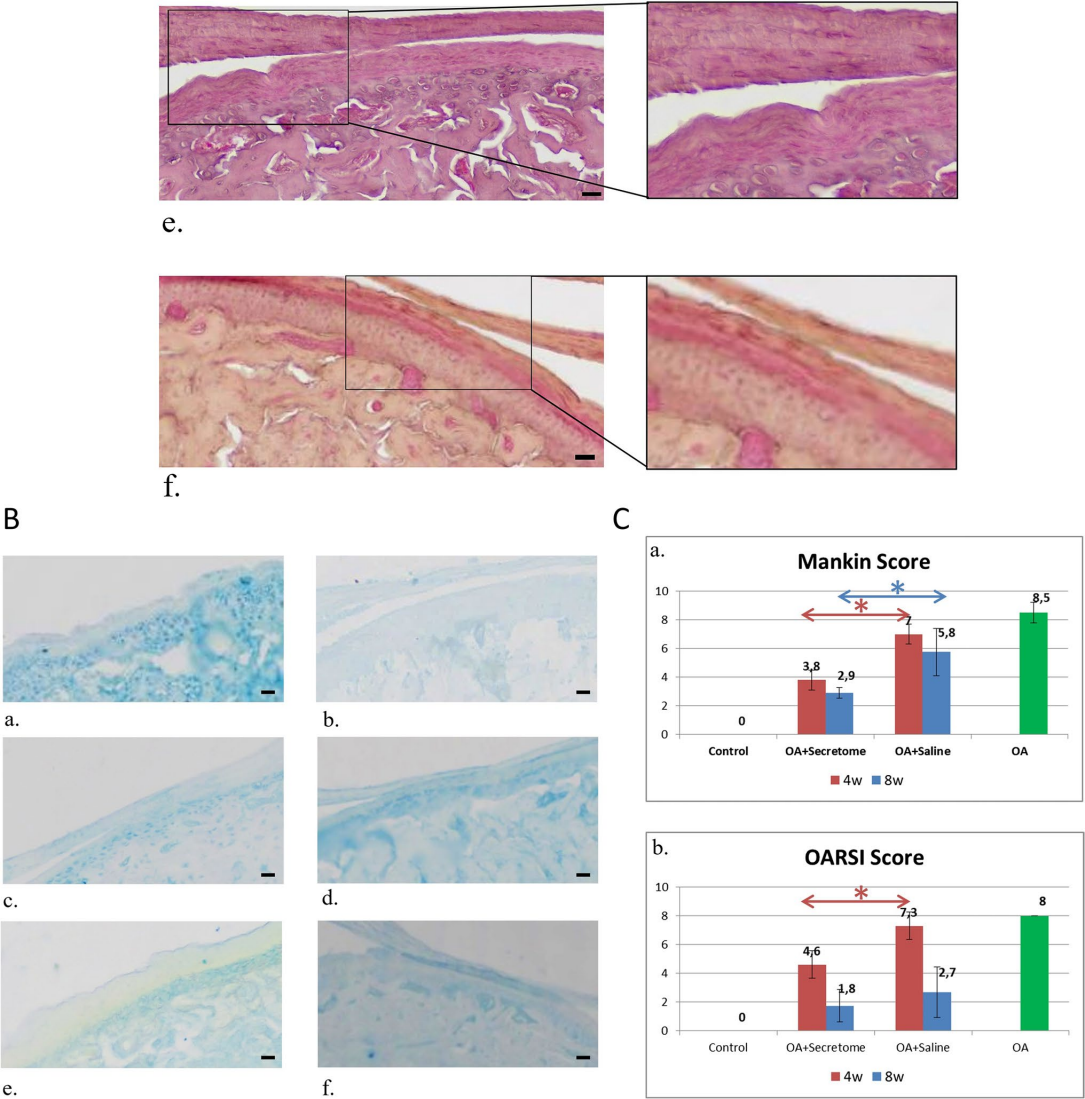
**A** H&E stain of histological sections. H&E stain of histological sections of control TMJ (a), TMJ with MIA-induced OA (b), saline-treated TMJ after 4wks of treatment (c), SECR-treated TMJ after 4wks of treatment (d), saline-treated TMJ after 8wks of treatment (e), and SECR-treated TMJ after 8wks of treatment (f) (40x, scale bar 20 μm). H&E stain of control TMJ, showing smooth articular surface and normal zonal architecture, with distinct layers, whereas sections of MIA-treated rats showing OA-like lesions with surface irregularities, bone erosion and bone marrow enlargement. The SECR-treated group showed improved cartilage surface, reduced bone erosion, and improved cartilage thickness (d) and the saline-treated group (*n* = 4) exhibited extensive bone erosion, surface irregularities with pannus formation (black arrows), along with diffuse hypercellularity and bone erosion (red arrows), and chondrocyte cloning or apoptosis (green arrow) (c). At 8wks post-treatment, the OA + SECR group exhibited pronounced improvement in cartilage surface integrity, cartilage thickness, cellularity, and subchondral bone integrity (f), the OA + Saline group exhibited discontinuity of cartilage surface, cell clustering close to areas of degeneration, and bone erosion (e). **B** AB stain of histological sections. AB stain of histological sections of control TMJ (a), TMJ with MIA-induced OA (b), SECR-treated TMJ after at 4 (c) and 8wks (d) showing hypercellularity and GAGs content comparable to the control TMJ, as it is depicted from the intense blue color, and saline-treated TMJ after at 4 (e) and 8wks (f) showing diffuse hypercellularity, and moderate reduction of GAGs compared to SECR-treated group, as it is depicted from the reduced blue color (10x, scale bar 20 μm). **C** Mankin scores (a) and OARSI scores (b) of the samples from the in vivo experiment. Mankin scores (a) and OARSI scores (b) from the TMJ samples following treatment at 4 and 8wks. Data represent mean ± SEM. *p < 0.05

Histological evaluation of the MIA-treated joints after 4wks of application supported radiographic data, indicating OA-like destruction of the cartilage and erosion of the subchondral bone. More specifically, OA-like lesions were observed, such as pannus and surface irregularities, diffuse hypercellularity of chondrocytes, chondrocyte cloning, apoptosis, bone erosion and bone marrow enlargement (Fig. 6A).

At 4wks after treatment, SECR-treated group (*n* = 5) showed improved cartilage surface, reduced bone erosion, and improved cartilage thickness (Fig. 6A). However, tidemark integrity was crossed by blood vessels in most samples, indicating increased angiogenesis (red asterisk) (Fig. 6A). However, that was not supported by the IHC analysis. In contrast, the saline-treated group (*n* = 4) exhibited extensive bone erosion, surface irregularities with pannus formation (black arrows), along with diffuse hypercellularity and bone erosion (red arrows), and chondrocyte cloning or apoptosis (green arrow) (Fig. 6A, Suppl. Fig. 6). Those observations were further supported by the grading through the Mankin's histological scoring system (Table 1) by three blinded independent observers, where the OA + SECR group had a Mankin score of 3.8 ± 0.8, as opposed to that in the OA + Saline group with a score of 7.0 ± 0.7 (*p* < *0.001*) (Fig. 6C). The Mankin score for the saline-treated group was similar to the OA-group i.e. positive control (no SS difference).

At 8wks post-treatment, the OA + SECR group (*n* = 6) exhibited pronounced improvement in cartilage surface integrity, cartilage thickness, cellularity, and subchondral bone integrity, and closely resembled the control TMJ condyle, indicating activation of reparative and regenerative procedures from the native joint-resident cells and SCs (Fig. 6A). On the other hand, the OA + Saline group (*n* = 8) exhibited discontinuity of cartilage surface, cell clustering close to areas of degeneration, and bone erosion (Fig. 6A). The Mankin score for the SECR -treated group at this time-point was 2.9 ± 0.4 as opposed to that in the saline-treated group which was 5.8 ± 1.7 (*p* < *0.001*) (Fig. 6C). In addition, 62% of the saline-treated TMJs exhibited TMJ disc thinning after 8wks, against only 14% of the SECR-treated group.

Alcian Blue (AB) staining revealed moderate to severe reduction of glycosaminoglycans (GAGs) in the saline-treated group compared to the SECR-treated group, which were similar to the naive control group (Fig. 6B).

Those results are further supported by the OARSI score (Table 2), as depicted in Fig. 6C. The OARSI score for the SECR-treated group at 4wks post-treatment was 4.6 ± 0.5 as opposed to that in the saline-treated group which was 7.3 ± 1.0 (*p* < *0.05*) (Fig. 6C). At 8wks post-treatment, there were no SS differences in the OARSI score between the two groups.

#### Immunohistochemical Analysis

**Fig. 7:**
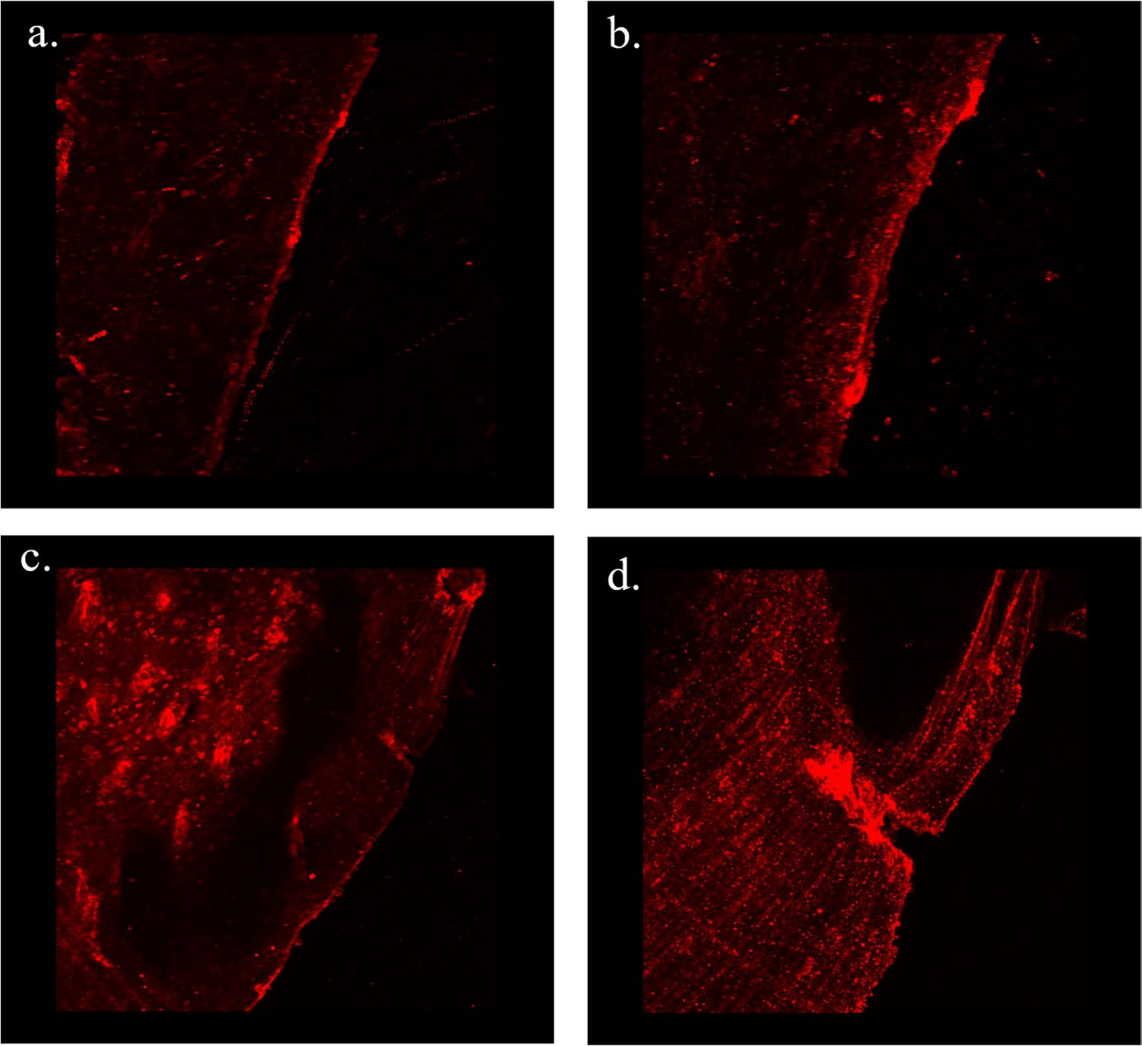
Immunohistochemical analysis. Positive staining for COLI after immunofluorescence labeling for SECR-treated TMJ at 8wks post treatment (**a**, × 10), exhibiting relatively smooth, healthy surface (**b**, × 40), compared to saline-treated TMJ (**c**, × 10), that presented extensive, in-depth lesions, which stained positive for COLI, but failed to heal properly after 8wks of treatment (**d**, × 40)

At 8wks post-treatment, SECR-treated group exhibited positive staining for COLI on the articular surface, with focal localization in two spots, where it could be OA-lesions that healed with increased production and deposition of COLI (Fig. 7a–b). On the other hand, saline-treated group presented extensive, in-depth lesions, that stained positive for COLI, but failed to heal properly after 8wks of treatment (Fig. 7c–d). IHC analysis for VEGF was not successful, while IHC analysis for von Willebrand factor showed non-evaluable differences between the different groups (data not shown).

## Discussion

To the best of our knowledge, this is the first study to administer an extensively analyzed and tailored secretome of dental origin in vivo for management of TMJ-OA. Collectively, our findings demonstrated that the SECR suppressed pain and reduced joint inflammation, enhanced ECM and subchondral bone repair and regeneration, thus alleviating TMJ degeneration. The SECR condition chosen was the most promising in terms of anti-inflammatory, regenerative and chondrogenic action in the local environment of the TMJ. This was supported by its enrichment in proteins with anti-inflammatory properties, proteins involved in extracellular matrix formation, cellular response to stress and detoxification of ROS.

In a previous study, SECR samples were prepared under various pre-conditioning methods (normoxia or hypoxia, with or without stimulation with TNF-α), analyzed via LC–MS/MS based proteomic analysis, and validated through the same in vitro inflammation model of LPS induced RAW 246.7 macrophages [16]. The SECR collected under normoxic conditions was pronouncedly enriched with proteins involved in pathways related to “ECM organization”, “cellular response to hypoxia”, and “IL signaling”, proteins with anti-inflammatory, anti-oxidant, and regenerative properties [16]. Extensive proteomic analysis and in vitro validation model was performed, and based on this analysis it was indicated that further tuning of the DPSCs preconditioning is required, in order to modulate the composition of the produced SECR. A complementary modification on the preconditioning method was applied in the present study to further improve the secretome composition [30, 31]. As a result, the hypoxic preconditioning was modified from 2 to 5%, and the time exposed to TNF-α from 2 to 24 h. The new condition (5% Hypoxia + TNF-a) for secretome collection that was used in the present study proved to have similar anti-inflammatory properties (based on the in vitro validation model) with the best (out of the four) secretome conditions (Normoxia) that was shown in the previous report to have the more pronounced anti-inflammatory profile. Besides, this new collection condition was shown to exhibit reduced cytotoxicity, thus emerging as the most promising secretome composition for in vivo application and future therapeutic use. The proteomic comparison between secretome and lysate LC/MS data was performed to validate whether these proteins have been enriched in the secretome or not. This point is highly important to identify truly secreted proteins from those proteins that might be present due to unspecific cell lysis. It is therefore intended to delve deeper into the mechanism (how the preconditioning affects protein secretion) and not to highlight the therapeutic effect. Qualitative analysis between secretome Normox (the optimum from the previous analysis) and SECR 5% Hypoxia + TNF-a (new condition) showed that 3 proteins were present in the SECR 5%Hypoxia + TNF-α and not in secretome Normox, COL6A3, periostin, and ACTN1. Quantitative differences among the proteins that are present in both Secretome conditions might be related to the different potential of each secretome. Οnly the optimum condition was chosen to be applied in vivo for ethical reasons, in order to be in line with the 3R’s, which serve as a framework for promoting the ethical use of animals in research while striving to minimize their suffering and maximize the scientific value of the experiments. The proteomic analysis of the new condition was performed after in vivo application to aid in the interpretation of the therapeutic action of secretome. SECR was further validated in the in vivo experimental model of rat TMJ-OA. The results clearly support that the optimized SECR has anti-inflammatory and regenerative potential and can be therefore proposed for further clinical validation as a therapeutic module of TMJ-OA in humans. This is mainly supported by its ability to reduce local pain and induce repair and regeneration via the attenuation of inflammation, enhancement of ECM repair and re-establishment of tissue homeostasis, as collectively shown by the clinical, radiographical and histological data.

Handling, treatment administration and clinical measurements were well-tolerated by the rats. Treated animals had no adverse effects and did not suffer during the experimental protocol. In addition, there were no adverse immune reactions from the use of human SECR in fully immunocompetent rats, highlighting the great potential of the applied protocol to be used in the future as a cell-free treatment for TMJ-OA management.

The first step of this in vivo model was to induce the experimental TMJ-OA. Drug induced TMJOA models, such as the one used in our study, while it exhibits a pathogenic mechanism that differs from human TMJOA, it has the advantages of ease of induction, produced small neglectable trauma, and is reproducible [32]. Additionally, by altering medication concentration, it is possible to control the rate of disease progression and the severity of joint lesions, providing an acute disease model for the creation of short-term studies [32, 33]. It was well-established through clinical, radiographic, and histological data that TMJ-OA was induced via the administration of MIA. Previous studies have shown that MIA injection into the upper compartment of the TMJ can successfully induced OA-like lesions through chondrocyte apoptosis and disturbance of cartilage and subchondral bone metabolism [33]. Initial response to MIA seems to be an increased inflammatory response, presence of synovitis, which results in hyperalgesia during the first weeks after administration. The initial inflammatory reaction is followed by distinct cartilage destruction and subchondral bone erosion. Following alleviation of inflammation, cartilage surface is undergoing fibrotic transformation, while sclerotic changes are observed in the subchondral bone [33]. Those sclerotic changes that are part of the reparative process observed in the TMJ condyle after the resolution of the initial inflammatory reaction are manifested as an unusual hardening or thickening of the bone and can be identified via the increased Tb.Th values, that became apparent 8wks post-treatment in both groups (saline-treated and SECR-treated).

Clinical evaluation revealed that treatment with SECR alleviated inflammation, reducing the sings of TMJ-OA, such as pain sensitivity and TMJ function (through food intake and weight measurements), to values like the naive control. TMJ-OA is a debilitating condition characterized by low-grade inflammation, and cartilage destruction attributed to an imbalance between the catabolic and the anabolic process in the ECM. Solid evidence highlights the importance of innate immune response and its “break-down” in the progression of OA [34]. Our results indicate that SECR is enriched in proteins related to innate immune response. This suppression of inflammation is consistent with the robust immunomodulatory action observed by the SECR via the in vitro validation model of the cultured LPS-induced RAW 246.7 macrophages, through the decrease of macrophages proliferation along with the down-regulation of the expression of pro-inflammatory genes, such as MMP-3, MMP-8, MMP-9, MMP-13, MCP-1, and IL-6. Simultaneously, macrophages maintained an increased expression of the anti-inflammatory cytokine IL-10. LPS exposure is associated with M1 polarization of macrophages. Further exposure to SECR seems to shift from M1 to M2 polarization, involved in resolving inflammation, promoting tissue healing, and suppressing excessive immune responses. IL-10 is a key cytokine involved in the M2 polarization process. IL-10 is primarily produced by M2 macrophages themselves and plays a crucial role in promoting and sustaining the M2 phenotype [35]. Increased IL-10 expression in LPS-DMEM samples might be related to the presence of glucose in the fresh medium (high glucose DMEM) compared to the SECR, that has been collected after 48 h in culture. There is evidence that changes in glucose metabolism influences macrophage polarization [36].

Radiographic evaluation has become indispensable during diagnostic evaluation of patients with TMJ diseases, including OA and conditions affecting articular surfaces, with Cone Beam / Computed Tomography (CB/CT) emerging as a powerful tool [37]. However, Micro-Computed Tomography (μCT) was chosen instead of CB/CT, being considered the “gold standard” for bone architecture and morphology evaluation in rats and mice in in vivo studies, as it produces high-resolution 3D images for precise data acquisition for the analysis of the bone structure, quality, and architecture [38] along with the potential of 3D measurements of the trabecular morphology, such as the trabecular thickness and trabecular separation. Application of μCT also allows a parallel to the histological analysis investigation. It is also a non-destructive technique; hence the same samples can be further used for a different analysis, which complies with the 3Rs’ rule for animals in research [11]. Finally, μCT analysis enables the qualitative and quantitative evaluation of bone mineralization [11].

Radiographic results from 4wk post-treatment showed that the OA + SECR group exhibited Tb.S values close to the control group, as well as substantial increase in mean density over the OA group, illustrating the repairing processes that have taken place. The inability to reach statistical significance in the results regarding the CT analysis 8 wk post treatment may be related to the time-point of assessment, since the reparative process regarding the course of treatment might have taken place earlier, and innate healing process might have taken place. Following alleviation of inflammation, cartilage surface is undergoing fibrotic transformation, while sclerotic changes are observed in the subchondral bone according to Wang et al. 2012, following MIA application [33]. Trabecular bone separation (Tb.S) is an index of bone microarchitecture regarding the main diameter of the cavities containing the bone marrow. Those results are more promising when compared with those from the study of Zhang et al., where significant differences in the bone parameters were observed only at 8wk post-treatment [10]. More specifically, it was found that bone structure parameters (BV/TV, Tb.Th, Tb. S and Tb.N) in the Exos-treated group were similar to those in the sham group, while the PBS-treated group exhibited deterioration in the same parameters relative to the sham group [10].

Histological evaluation revealed that by the end of the 8wk treatment, SECR -treated TMJs showed remarkable reconstruction of the damaged condylar structure, matrix deposition and subchondral bone integrity that were comparable to that of the naive control group. Several proteins have been previously identified in the SECR that participate in tissue homeostasis, such as TGFβ1 and TGFβ2, collagens, including COL1A1, COL1A2, and COL2A1, and metalloprotease inhibitors, including TIMP1 and TIMP2 [16]. The present analysis of SECR also revealed the presence of proteins related to ECM organization, such as collagens (including COL1A1, COL1A2, COL5A1, and COL6A1), biglycan, fibronectin, and decorin. ECM degradation through increased catabolic process, reduced cell proliferation and chondrocyte apoptosis are the cardinal features in the development and progression of TMJ-OA [1]. The catabolic process is mainly implemented through the increased expression and function of the MMPs and ADAMTS that degrade ECM components, such collagens and proteoglycans [39]. Albeit MMPs are residing in the healthy TMJ participating in tissue homeostasis and remodeling, while overexpression makes them part of the destructive disease process [40]. In contrast, IL-10 plays a fundamental role in preventing inflammatory and autoimmune pathologies [41]. The chosen secretome condition had the most pronounced effect in down-regulating the expression of the pro-inflammatory markers, while up-regulating the mRNA expression of IL-10. It also presented low values at the LDH assay, indicating absence of cytotoxicity caused by the pre-conditioning protocol applied.

Regarding the pathways involved in the therapeutic action of the SECR, it seems that the main mechanism is through the inhibition of the STAT1 pathway [42]. Local injection of DPSCs in a murine model of experimentally-induced TMJ-OA relieved hyperalgesia and synovial inflammation, attenuated ECM degradation, and induced bone regeneration [42]. It was further showed that MMP3 and MMP13 expressions were regulated by STAT1, and application of STAT1-specific inhibitor could block the expression of those proteins.

Previous studies that used SCs derived SECR supported its anti-inflammatory effect in animal models of experimentally-induced arthritis [43–45]. In an anti-collagen type II antibody-induced arthritis, application of Bone Marrow SC-derived SECR (BMSC-SECR) and SCs from human exfoliated deciduous teeth-derived SECR (SHED-SECR) led to a significantly reduced level of pro-inflammatory cytokine gene expression [45]. Moreover, in a rat model of antigen-induced arthritis, application of BMSC-SECR resulted in edema and thermal hyperalgesia reduction, reducing also the serum levels of TNF-α [43]. Mao et al. showed that cartilage development and homeostasis was regulated through the Wnt5a signaling pathway [46]. Additionally, through the overexpression of miR-140-5p, MSC-Exos exhibited an enhanced protective effect against OA by promoting chondrocyte proliferation and migration and inhibiting chondrocyte hypertrophy via the Wnt5a/NFkB and Wnt5b/JNK pathways [47, 48], while their application in a rat osteochondral defect model resulted in M2 macrophage infiltration along with decrease in M1 macrophages and inflammatory cytokines [49].

MSC-SECR has also exhibited reparative and regenerative abilities. MSC-SECR administration has shown positive results in the management of degenerative conditions through the activation of the endogenous healing potential via the administration of trophic factors that suppress local and systemic immune response [50]. MSC-SECR administration could alleviate OA through balancing the MMP-13 to TIMP-1 ratio in cartilage, inhibiting chondrocyte apoptosis, and enhancing autophagy [51]. SECR application seems also to have a protective effect against cartilage damage, and subchondral bone architecture by preventing chondrocyte apoptosis, reducing ECM proteolysis, repairing the damaged cartilage with hyaline cartilage instead of fibrocartilaginous tissue, and reducing osteocyte apoptosis and osteoclastic activity [37, 44, 45, 51, 52]. Native joint-resident SCs are abundant in the TMJ, including cartilage, bone marrow, synovium, joint adipose, and synovial fluid [53]. Those cells have a protective role in cases of degenerative disease and are probably the cells that are activated by the factors present in the secretome, restoring tissue homeostasis and repairing cartilage damage. Synovial MSCs exhibit superior chondrogenic ability compared with other native joint-resident SCs [38, 42, 54].

Several in vivo studies of experimentally-induced arthritis have shown that MSC-SECR/Exos/MVs application had positive results in terms of resolution of inflammation and cartilage regeneration [9]. However, most of them evaluated the application of SECR/Exos/MVs in models of knee OA, from which results and conclusions cannot be directly extrapolated to the TMJ-OA. There have been only two studies evaluating the application of SECR or Exos in experimental models of TMJ-OA. In the study by Zhang et al., MSC-Exos were used in a rat model of TMJ-OA [10]. Their proposed treatment had promising results, exhibiting pain alleviation, TMJ repair, similar to those demonstrated in the present study, and regeneration through adenosine activation of AKT, ERK and AMPK signaling [10]. In the study by Ogasara et al., SHED-SECR was administrated to a mechanical-stress induced model of experimental murine TMJ-OA, as opposed to the present chemically induced model [11]. SHED-SECR administration resulted in reduced pain, and improved cartilage surface and bone integrity, while also decreasing the expression of IL-1b, iNOS, and MMP-13 [11]. Even though our study uses dental-derived secretome, the same as Ogawasara et al., the cells used were different [11]. We used Dental pulp stem cells, whereas Ogawasara used human exfoliated deciduous teeth stem cells. In addition, secretome preparation was different. We applied preconditioning and concentrated the collected secretome. Additionally, we administrated the secretome intra-articularly, whereas Ogawasara et al. used intravenous administration [11]. Intra-articular administration of MSCs has been found more effective when compared to intravenous administration [55].

Oxidative stress has been recognized as a pivotal contributor factor in the progression of inflammatory and degenerative disease, such as TMJ-OA [1]. Oxidative stress is a result of the loss of balance between the production of reactive ROS as a response to external stimuli, and the capacity of the cell detoxification mechanisms to counteract ROS via specific mechanisms [56]. SECR analysis revealed that SECR samples were enriched with several proteins involved in cellular response to stress and proteins involved in detoxification of ROS, such as SOD2, Peroxiredoxin-1 (PRDX1), and Thioredoxin (TXN). SOD enzymes have been found to play an essential role in controlling ROS and are the major enzymatic antioxidant defense against inflammatory and degenerative diseases, such as TMJ-OA [57]. Therefore, increased levels of anti-oxidative and oxidoreductive proteins might have contributed to the therapeutic effect observed after the application of SECR.

In the times of extraordinary findings by James Webb, DPSC-S could probably be considered the liquid phase cell-free matter capable of breaking the symmetry of low OA inflammation[58] by complete covering asl liquid, in the tightest way, the irregular geometry of destructed TMJ surfaces, blocking the ROS, activating and harnessing resident SCs[59], dedifferentiating resident cells and busting proliferation[60], increasing by its administration synovial space and decreasing articular loading thus affecting cellular environment and kinetics[61], orchestrating the most effective upregulation and downregulation of pathways for pain relief and regeneration.

The ultimate clinical target of the present protocol is to develop an off-the-shelf fully characterized product that would be administered in any chronically inflamed joint, not necessarily the TMJ, and induce anti-inflammatory action along with repair. Cell-free therapeutic approach with the use of DPSC-SECR can address a wider range of patients, including those suffering from knee or hip OA, while offering the advantage of being inclusive, time- and cost-effective, have a low potential for toxicity and immunogenicity, and the product can be stored for longer periods via cryopreservation than MSCs.

## Conclusions

Collectively, from the results of the present study, it can be contemplated that the therapeutic effects of SECR application in the case of experimental model of TMJ-OA include inflammation alleviation through the reduction of macrophages, decrease in the expression of MMP-9, MMP-13, MCP-1, and increase in the expression/production of anti-inflammatory cytokines, such as IL-10, improvement on all the clinical parameters, resulting in improved food intake, weight, and pain suppression, as well as radiographically, on the trabecular bone thickness and bone mineral density. Histological sections indicated that SECR administration reduced inflammation, enhanced ECM and subchondral bone repair and regeneration through stimulating endogenous healing potential, thus alleviating TMJ degeneration.

Our data demonstrated that SECR treatment promoted the regeneration and repair of chemically induced TMJ-OA model suggesting that SECR has great potential to be a cell-free therapeutic approach for patients with TMJ-OA.

### Supplementary Information

Below is the link to the electronic supplementary material.

Supplementary file1 (DOCX 3337 KB)

## Data Availability

All data are available in the main text or the supplementary materials or can be made available after contact.
