# Supplementary material for: Managing Temporomandibular Joint Osteoarthritis by Dental Stem Cell Secretome
Source: Stem Cell Rev Rep. 2023 Sep 26;19(8):2957–79. doi: 10.1007/s12015-023-10628-9 (PMC10661765; doi:10.1007/s12015-023-10628-9)
Supplement: Supplementary file 1 — Supplementary file1 (DOCX 3337 KB) [file 12015_2023_10628_MOESM1_ESM.docx]

**Supplementary Material**

**Materials & Methods**

**Evaluation of adipogenic and osteogenic differentiation potential of DPSCs**

For the assessment of DPSCs adipogenic potential, cells from established cultures were seeded at 3 × 10^5^ cells/well in 6-well plates in CCM until reaching confluency. DPSCs

were then exposed to adipogenic medium (a-MEM CCM, supplemented with 0.5 mM isobutylmethylxanthine (IBMX), 0.5 mM hydrocortisone, 60 mM indomethacin and 5 mg/ml insulin, all from Sigma–Aldrich) for 21 days, followed by Oil-Red-O (Sigma–Aldrich) staining and visualization under light microscopy.

Additionally, for the assessment of osteogenic differentiation potential of DPSCs, seeded cells were cultured with osteoegenic medium () for 21 days, followed by Alizarin Red S (Sigma-Aldrich) staining and visualization under light microscopy.

**Sample size analysis**

The calculation of the sample size was performed with the "head withdrawal threshold" as the main variable and was based on the mathematical formula:

n= 2×(z1-a/2 + zβ )2×σ2×(1+(m-1)×ρ)/(m×Δ2)

= 2×(z1-a/2 + zβ )2×(σ2/ Δ2)×(1+(m-1)×ρ)/m (1)

presented by H. Brown & R. Prescott [21]. It applies to designs with repeated measurements, where the dependent variable follows a normal distribution and the sphericity assumption is applied [21].

In formula (1) the ratio Δ/σ corresponds to the ratio of the mean difference between the experimental and control groups to the standard deviation of the measurements (σ2 is the between-patient variation). This ratio (inverse of the coefficient of variation) devoid of units of measurement was approximated by the article by Wu et al. 2010 [22] and was used for the initial estimation of the study sample size. More precisely, from a bar chart that depicts the mean and standard error of the 'head withdrawal threshold' variable, it was estimated that after the experimental OA, an average decrease of 21.5 points was observed while the standard deviation was calculated to be √5. × 5 = 11.18, where value 5 corresponds to the standard error (SE) of the mean and √5 is the square root of the number of laboratory animals (sample size in reference article [22] which gave estimates after OA challenge). Using the previous values, Δ / σ = 1.9 was calculated and this value was used in formula (1). In addition, it was assumed that there would be m = 4 repetitive measurements (2 measurement times, before OA application and after treatment completion and two left and right side measurements of each animal, one of which will be used as control). The probability of a Type I error (level of statistical significance) was set at alpha = 0.05 (hence z1-a / 2 = 1.964), study power equal to 80% (hence zb = 0.842), and the linear correlation coefficient between replicates, equal to p = 0.5. So formula (1) gave:

n = 2 × (1.96 + 0.842) 2 × (1.9) 2 × (1 + 3 × 0.5) / 4 = 19.1

that is, 19 laboratory animals per group. However, considering that there may be up to 10% loss, attrition rate = 10%, the final number per group is set to:

n = 19 × 100 / (100-10) = 21.

Therefore, the initial estimate is that 21 laboratory animals will be needed so that the study has an 80% chance of showing a statistically significant difference between the experimental and the control group, almost equal to the improvement near complete recovery.

The randomization of the laboratory animals to the control joint will be done by block randomization.

**Results**

**Adipogenic and osteogenic differentiation of DSPCs**

Adipogenic differentiation of DPSCs was shown by formation of Oil-Red-O positive, lipid-rich vacuoles (Supl. Fig. 1a), while osteogenic differentiation of DPSCs was shown by calcified deposits as shown by the positive Alizarine Red S staining (Supl. Fig. 1b).

**Supplementary Figures**


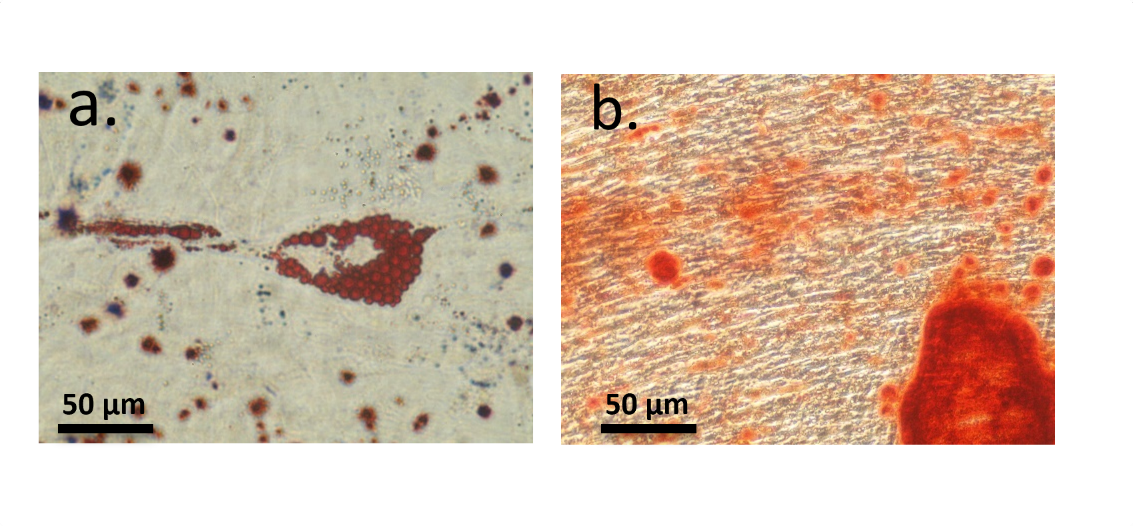


**Supplementary Fig. 1** Optical microscopy image of Oil-Red-O (a) and Alizarin Red S (b) staining of DPSCs in 2D TCPS surfaces when exposed to adipogenic and osteogenic medium, respectively, for 21 days.


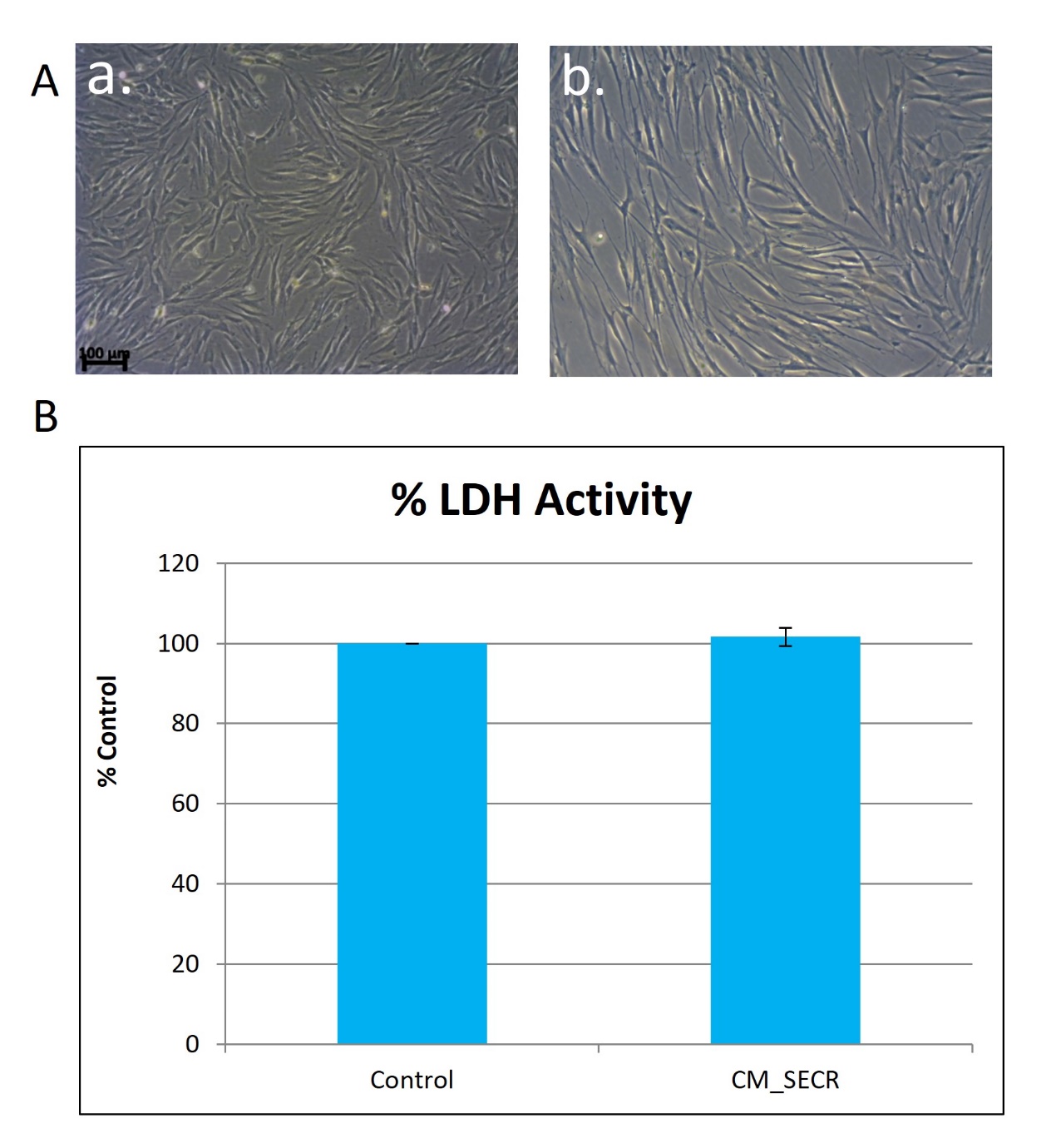


**Supplementary Fig. 2A** Morphological assessment of DPSCs after the application of preconditioning. (a) Control DPSCs and (b) DPSCs preconditioned in 5% hypoxia and TNF-a. TNF-a preconditioning for 24h and 5% hypoxia for 72h affects the morphology of the cells, since cells adopt an elongated morphology. **2B** Cytotoxicity assessment of the preconditioning methods on DPSCs.


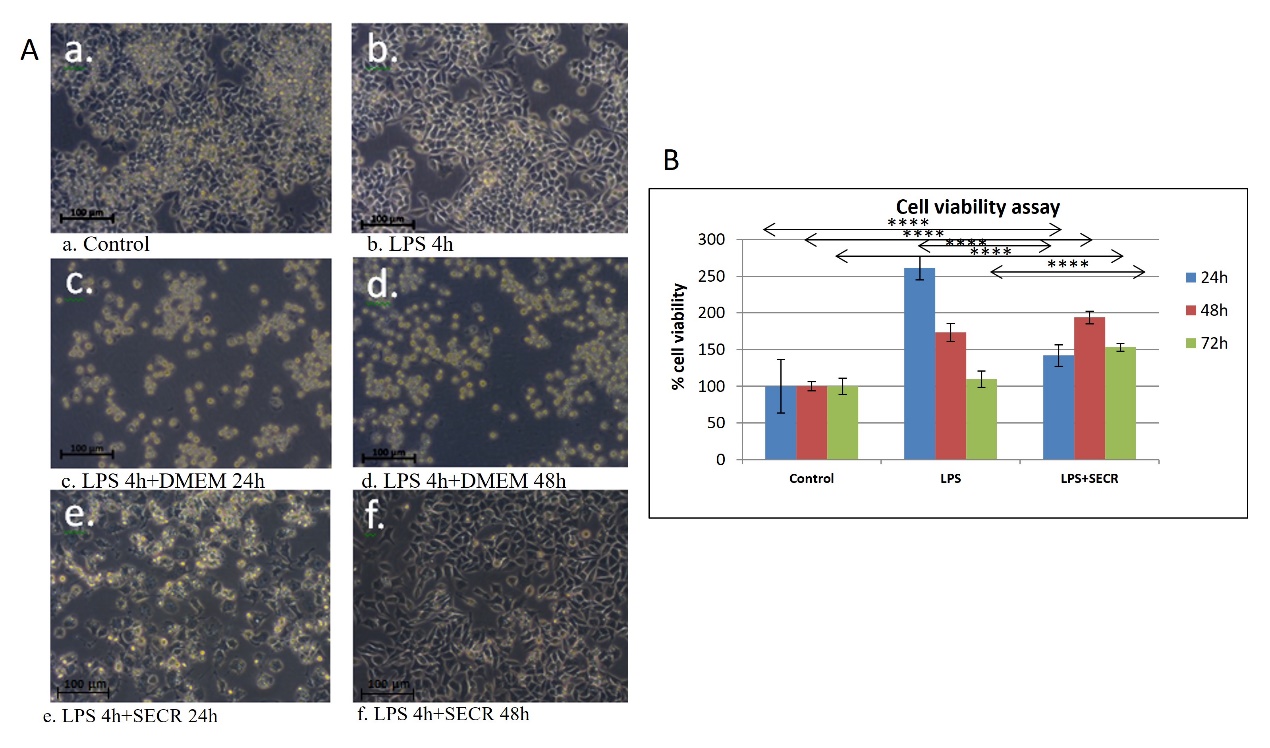


**Supplementary Fig. 3A** Morphological assessment of RAW cells after the application of LPS and CM. Normal RAW cells display an irregular form with pseudopodia (a). LPS application (b) affected the morphology of RAW cells, which were transformed into a totally circular form after 24 and 48 h of the LPS application (c and d). This effect was partly reversed by the application of CM_5%Hyp+TNF-a after 24h (e), and totally reversed after 48h in culture with CM_5%Hyp+TNF-a (f). **3B** Viability/proliferation of RAW cells after the application of LPS and CM. Addition of CM_5%Hyp+TNF-a had a stimulatory effect on RAW cell viability/proliferation after all the time-points assessed, resulting in significant increase in cell viability compared to control RAW cells (p<0.0001). This stimulatory effect was significant against LPS (p<0.0001) after 72h. ****p<0.0001.


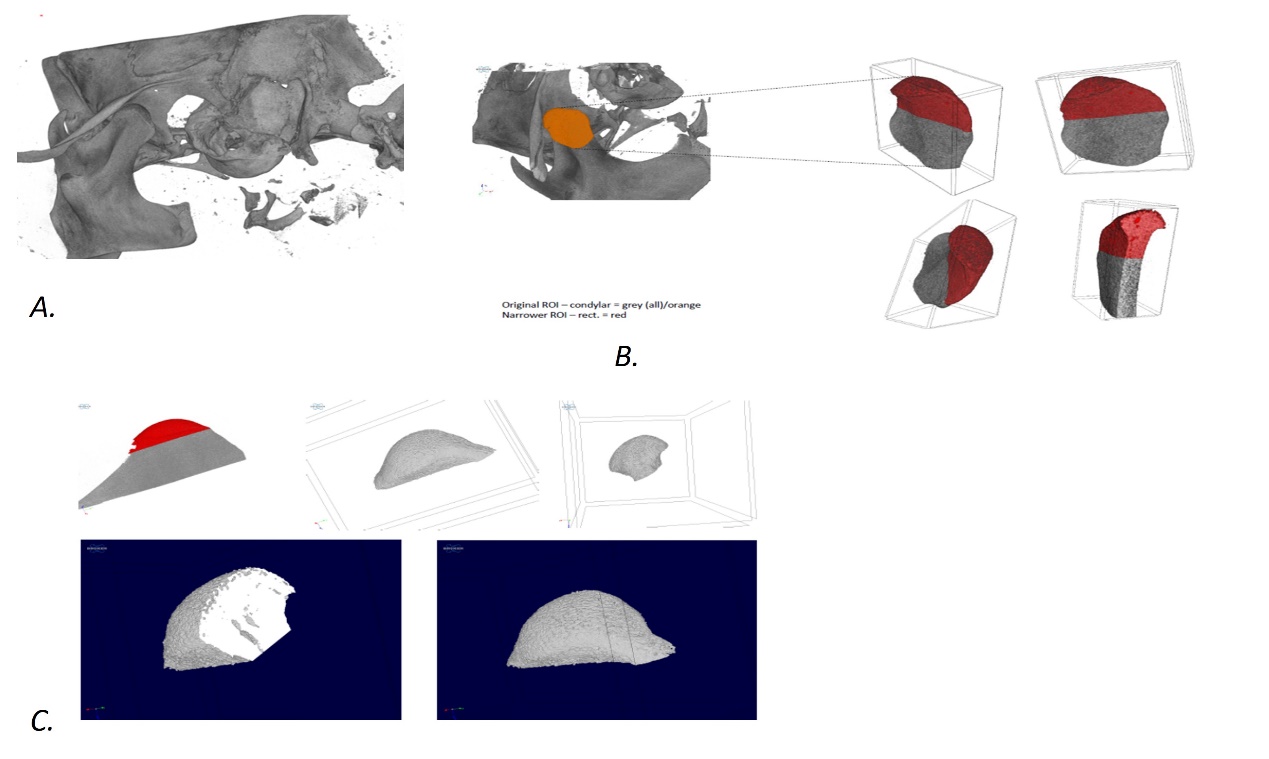


**Supplementary Fig. 4A** 3D model of a rat TMJ scanned in a Brucker μCT scanner. **4B** Initial attepts to select the region of interest (ROI). **4C** Final ROI used in the study.


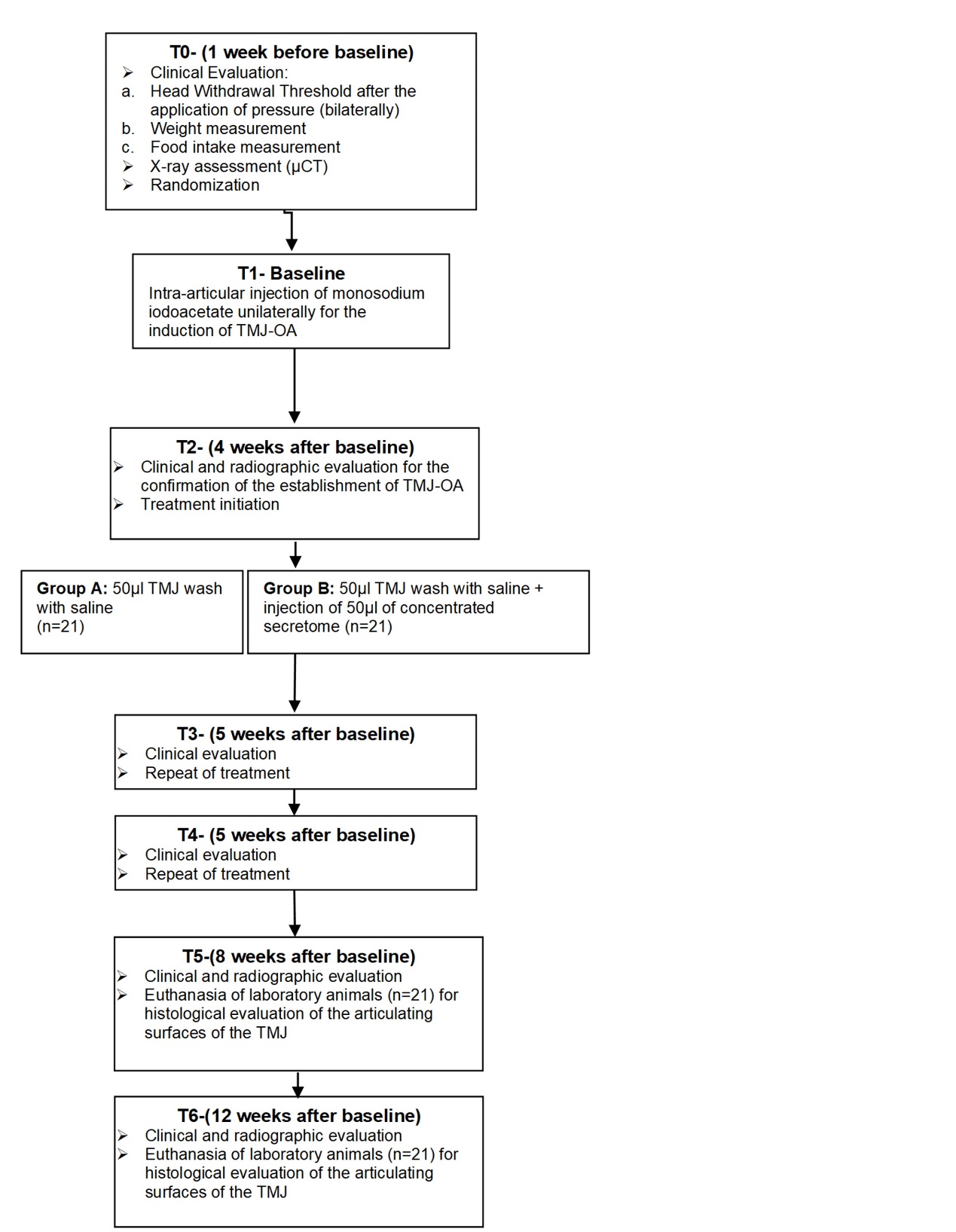


**Supplementary Fig. 5.** Timeline of experimental procedures of the in vivo study.


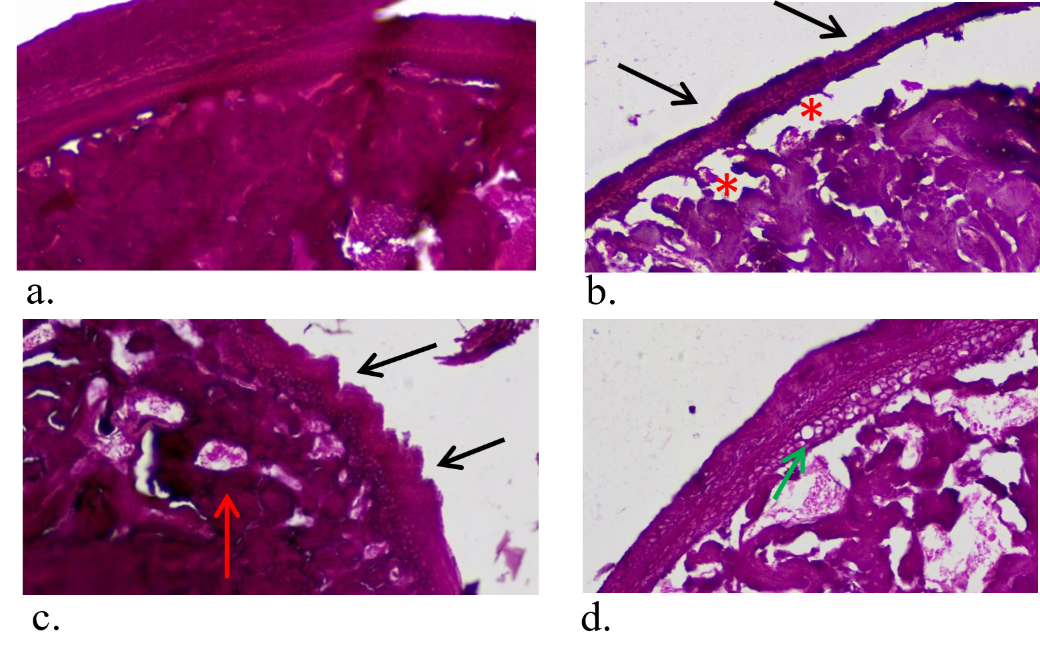


**Supplementary Fig. 6. H&E stain of histological sections.** H&E stain of histological sections of saline-treated TMJ after 4wks of treatment (a, b), SECR-treated TMJ after 4wks of treatment (c, d)(40x, scale bar 20μm). The SECR-treated group showed improved cartilage surface, reduced bone erosion, and improved cartilage thickness (a, b) and the saline-treated group (n=4) exhibited extensive bone erosion, surface irregularities with pannus formation (black arrows), along with diffuse hypercellularity and bone erosion (red arrows), and chondrocyte cloning or apoptosis (green arrow) (c, d).
